# Economic Policy Uncertainty and Health: Empirical Evidence from the MIDAS Model

**DOI:** 10.3390/healthcare14111460

**Published:** 2026-05-25

**Authors:** Min Lin, Jipeng Fei

**Affiliations:** Bay Area International Business School, Beijing Normal University, Zhuhai 519087, China; minlin@bnu.edu.cn

**Keywords:** MIDAS model, mortality rates, economic policy uncertainty, lifestyle behaviors

## Abstract

**Background/Objectives**: While the health effects of economic fluctuations are well-documented, the role of policy-related uncertainty remains underexplored. The objective of this study is to examine the association between economic policy uncertainty (EPU) and mortality. Furthermore, we investigate whether changes in lifestyle behaviors are associated with EPU and may help shed light on the relationship between EPU and health outcomes. **Methods**: We utilize a mixed data sampling (MIDAS) framework to analyze US state-level data from 2009 to 2020. The model controls for unemployment, income, demographic characteristics, as well as state and year fixed effects. This approach enables the incorporation of high-frequency uncertainty measures to capture dynamic mortality responses. **Results**: The results indicate a statistically significant inverse association between EPU and total mortality. The association is negative across both genders, with a stronger effect observed among males. Across age cohorts, the retirement-age group exhibits the highest sensitivity. In terms of cause-specific mortality, EPU is positively associated with mortality from respiratory diseases and suicide, while it is negatively associated with mortality from homicide, accidents, and pneumonia and influenza. In addition, EPU is significantly associated with a lower prevalence of current drinking and smoking, a higher likelihood of being in a healthy weight range, improved self-reported health, and reduced time spent traveling. **Conclusions**: The findings suggest heterogeneous associations between EPU and mortality outcomes across demographic groups and causes of death, highlighting the complex and multifaceted nature of the relationship between policy-related uncertainty and population health rather than a uniform response across health outcomes.

## 1. Introduction

It is widely recognized that one objectives of economic policy is to maintain and improve population health. However, the implementation of such policies is often associated with uncertainty regarding their economic consequences. The 2016 US presidential election provides a notable example of policy-induced economic uncertainty. According to [1], this election generated increased uncertainty across various sectors, including healthcare, due to concerns about the implementation of new policies and the potential revocation of existing policies under the incoming Trump administration.

Evidence has shown that economic policy uncertainty has emerged as a critical factor associated with a wide range of health outcomes [2,3,4,5,6,7,8,9,10,11]. Understanding the relationship between economic policy uncertainty and health outcomes is imperative, given its potential implications for public health and policy design. Despite its importance, the specific pathways through which economic policy uncertainty is associated with health outcomes remain underexplored in the literature. This paper contributes to the literature by investigating how economic policy uncertainty is related to mortality through various lifestyle behaviors, leveraging an innovative methodological approach to provide new insights.

This paper employs the Economic Policy Uncertainty (EPU) index developed by Baker et al. [12] as a proxy for uncertainty regarding the scope and direction of future economic policy. We investigate sex-specific, age-specific, and cause-specific mortality rates to capture heterogeneous associations with EPU. Specifically, four age groups are examined: 15–34, 35–54, 55–64, and ≥65-year-olds, and nine specific causes are studied: respiratory disease, cardiovascular disease, ischemic heart disease, cerebrovascular disease, cancer, suicide, homicide, accident, and pneumonia and influenza.

Incorporating the EPU index into the mortality model of Ruhm [13], our analysis yields several significant findings. First, there is an inverse association between EPU and total mortality rates, with a one-point increase in EPU associated with a reduction of 0.0372 deaths per 100,000 population. This finding is consistent with the cross-national evidence provided by Huang et al. [11], who similarly document a negative association between EPU and mortality. Second, EPU is inversely associated with mortality rates across both genders, with the association being more pronounced for males than for females. Third, across age cohorts, the most notable variation in mortality is observed among the retirement-age population, highlighting greater sensitivity of older adults to economic policy uncertainty, consistent with Huang et al. [11]. Lastly, cause-specific analysis indicates heterogeneous associations between EPU and mortality outcomes. Specifically, higher EPU is associated with increased mortality from respiratory diseases and suicide at the 5% significance level. Conversely, it is associated with lower mortality from homicide, accidents, and communicable diseases. To assess robustness, we implement the Oster bound test to evaluate potential sensitivity to unobserved selection. The results indicate that our baseline estimates are robust to omitted variable bias, indicating that the observed association between EPU and mortality is not fully driven by unobserved confounding.

We further examine potential mechanisms linking EPU to health outcomes by focusing on lifestyle-related behaviors. Previous studies on the relationship between economic conditions and health have shown that changes in health behaviors may contribute to procyclical fluctuations in mortality [13,14]. Building on these insights, we investigate whether similar behavioral adjustments are associated with EPU. Using data from the Behavioral Risk Factor Surveillance System (BRFSS) and the American Time Use Survey (ATUS), we analyze a range of health-related behaviors, including alcohol and tobacco consumption, body weight status, self-reported health, mental and physical distress, and time allocation patterns such as travel, socializing, sports, and personal medical care.

Furthermore, the methodological innovation of this study lies in the application of a panel-MIDAS model to examine the relationship between EPU and health outcomes. Unlike conventional approaches in macroeconomics and health research that typically assume variables observed at the same frequency, such as fixed-effect models [4,7,8,11,13,15], error correlation models [16,17,18,19], VAR models [20,21,22], and standard OLS models [2,3,5,9], our dataset comprises data observed at three different frequencies (monthly, quarterly, and annual). To fully exploit the information contained in high-frequency data, we use the MIDAS model [23], which allows regression analysis using variables sampled at mixed frequencies. Additionally, given that the association between EPU and mortality may unfold over time, the MIDAS specification is particularly suitable as it accommodates distributed lag structures of monthly EPU over several years. This feature allows the analysis to capture longer-run associations between economic policy uncertainty and health outcomes.

The rest of this paper is organized as follows. Section 2 reviews the related literature. Section 3 provides a conceptual model outlining the potential association between EPU and health outcomes. Section 4 and Section 5 present the estimation of the association between EPU and mortality and lifestyle behaviors, respectively, including data sources, empirical methodologies, and estimation results. Section 6 presents robustness analyses to further validate our findings, and the last section concludes.

## 2. Literature Review

### 2.1. The Association Between Economic Policy Uncertainty and Health Outcomes

Research on the relationship between macroeconomic conditions and health has received considerable attention since the pioneering work of Brenner [17,24,25,26,27]. Using aggregate time-series data, Brenner uncovers a counter-cyclical pattern in mortality. This pattern has been further examined in subsequent studies, including Bianchi et al. [22], Gerdtham and Johannesson [28], Browning and Heinesen [29], McInerney and Mellor [30], and Laliotis and Stavropoulou [31]. However, Brenner’s findings have also been subject to criticism, as several studies fail to replicate these results across different countries and time periods [16,32,33,34,35].

Contrary to Brenner’s results, Ruhm [13] reports the opposite relationship. Using a panel data model, he finds that an increase in the unemployment rate is associated with a decline in total mortality of approximately 0.5% over the period 1972–1991 in the US. His subsequent work further supports the procyclicality of total mortality [36,37,38]. A large body of later research provides similar evidence across various countries, including Germany [39], Norway [40], Spain [41,42], Sweden [43], OECD countries [44], European countries [45], and Canada [46].

Beyond the well-documented relationship between economic conditions and health status, a growing body of research has examined the association between economic uncertainty and health outcomes. Much of this literature relies on proxies such as job insecurity [47,48,49,50,51] and financial insecurity [52,53,54] to capture uncertainty. For example, Ferrie et al. [47] use job insecurity to measure economic uncertainty and examine its association with health among British white-collar civil servants, showing that self-reported job insecurity is linked to poor self-rated health and minor psychiatric morbidity. Similarly, Bridges and Disney [52] utilize financial insecurity as a proxy and document a positive association between subjective measures of financial well-being and psychological well-being. Other studies employ alternative measures of uncertainty. Abdou et al. [55] use stock market volatility as an indicator of economic uncertainty and report a positive association with suicide rates in the US. Liu [56] uses the World Uncertainty Index (WUI) and finds that higher uncertainty is associated with longer life expectancy and lower mortality rates. In addition, a related strand of literature shows that major uncertainty-inducing events are associated with changes in mental health and well-being [57,58,59,60].

Focusing specifically on policy-related economic uncertainty, a growing body of literature examines its association with health outcomes, with particular attention to suicide. Using time-series data from 1960 to 2013 in the US, Antonakakis and Gupta [2] provide the first empirical evidence that higher economic policy uncertainty is associated with increased suicide rates among younger and older male groups, while the female population appears less responsive. This positive association between economic policy uncertainty and suicide has been further documented in short-run analyses for the UK [3] and the US [4], as well as in cross-country evidence reported by de Bruin et al. [61].

Recent scholarship has further identified asymmetries and temporal complexities in this relationship. Abdou et al. [5] indicate that unexpected increases in economic policy uncertainty are associated with higher suicide rates among prime-age males (25–64) and elderly females (65+), whereas unexpected decreases are not statistically associated with changes in suicide. Similarly, Claveria [62] finds that contemporaneous uncertainty may be associated with short-term declines in suicide, whereas lagged uncertainty is linked to subsequent increases. Furthermore, Er et al. [63] show that the association between economic policy uncertainty and suicide varies by national income levels, with stronger associations observed in high-income economies and no statistically meaningful association in middle- and low-income contexts. Beyond suicide, EPU has also been associated with other mortality outcomes, including road traffic mortality [7] and cardiovascular disease mortality [9,10].

Despite an increasing number of studies on specific causes of death, evidence on the association between economic policy uncertainty and total mortality remains limited. To the best of our knowledge, Huang et al. [11] provide the first systematic investigation of this relationship. Using a panel dataset of 17 countries from 1985 to 2018, their study demonstrates that higher levels of EPU are associated with lower mortality and better health outcomes, particularly among older adults aged 65 and above.

However, significant methodological and contextual limitations remain in the study by Huang et al. [11]. While their work offers a valuable cross-national perspective, their reliance on annual aggregate data may inadvertently mask the immediate, intra-year dynamic responses to policy shocks. Furthermore, the use of national-level data often smooths over the substantial sub-national heterogeneity found in large economies like the US. Our paper addresses these limitations by investigating the association between EPU and mortality at the US state level through an innovative panel-MIDAS framework. By integrating high-frequency monthly EPU fluctuations with annual mortality data, we are able to capture the temporal dynamics that conventional annual models overlook. In doing so, this study provides a more granular and robust understanding of how policy-related uncertainty is associated with public health across the life cycle.

### 2.2. The Association Between Economic Policy Uncertainty and Behavioral Outcomes

In Section 3, we outline four potential mechanisms through which EPU is associated with individuals’ lifestyle behaviors, comprising one direct channel and three indirect channels. The direct channel primarily operates through mental health, referred to as the psychological pathway. The three indirect channels encompass reduced household spending, time allocation, and decreased travel and mobility. In this subsection, we review the relevant literature on the relationship between economic policy uncertainty and economic outcomes.

From a macroeconomic perspective, uncertainty is closely related to the inability to assess the economy’s current state and unpredictable future economic outlook. Elevated uncertainty matters due to its profound and widespread impact on both consumers and firms. The existing literature suggests that uncertainty is associated with reductions in economic activity, including consumption spending [64,65,66], investment and hiring [67,68,69,70], and trade [71,72,73]. For example, Carroll [64] demonstrates that consumers facing greater income uncertainty tend to reduce consumption due to stronger precautionary saving motives. Bloom [70], utilizing stock market volatility as a proxy for uncertainty, provides evidence that heightened uncertainty encourages firms to postpone or cancel investment and hiring decisions. Similarly, Baker et al. [12] find that economic policy uncertainty leads firms to delay investment and reduce employment. Handley and Limão [72] show that trade policy uncertainty significantly reduces trade flows.

Beyond aggregate macroeconomic indicators, economic policy uncertainty also influences individual health-related behaviors [11,74]. For example, Kalcheva et al. [74] examine how drinking and smoking behaviors respond to changes in economic policy uncertainty. Their results indicate that higher economic policy uncertainty is associated with lower levels of impulse control and adverse lifestyle choices, such as higher rates of alcohol consumption, greater drinking intensity, and a higher prevalence of binge drinking. In contrast, Huang et al. [11] indicate that higher EPU is associated with a reallocation of time toward self-care and leisure activities, healthier behaviors, and a reduction in labor supply.

Another strand of the literature examines the association between economic uncertainty shocks and travel behavior. During periods of heightened economic uncertainty, households may be inclined to reduce and postpone tourism-related expenditures, as travel spending is particularly sensitive to perceptions of instability and uncertainty in the economy [75]. Demir and Gozgor [76] further emphasize that increases in economic policy uncertainty can result in the postponement or cancellation of travel plans due to concerns about security, safety, and stability, thereby discouraging overseas travel.

## 3. Conceptual Model

Unlike unemployment or realized economic downturns, economic policy uncertainty does not necessarily translate into immediate changes in individuals’ financial conditions [9,10]. Rather, it can be viewed as a forward-looking stressor that may shape expectations about future economic conditions. As a result, behavioral responses are more likely to reflect anticipatory or precautionary adjustments, rather than reactions to realized income or employment shocks. For example, individuals may increase labor supply in anticipation of future instability or reduce consumption and increase savings as a precautionary response. This distinction also differentiates EPU from unemployment. As emphasized by Ruhm [13], mechanisms linking economic conditions to health—such as changes in the opportunity cost of time due to job loss or exposure to hazardous working conditions—are typically associated with realized labor market fluctuations. In contrast, in the absence of changes in unemployment, these channels may be less directly affected by EPU. Instead, EPU may operate through expectation-driven and behavioral pathways that differ from those associated with actual job loss.

Overall, while both EPU and unemployment may be associated with changes in health-related behaviors and outcomes, the underlying mechanisms are likely to differ. In what follows, we outline four potential channels through which EPU may be associated with lifestyle behaviors and health outcomes.

### 3.1. Decreased Household Spending Channel

One potential mechanism is that higher uncertainty is associated with increased precautionary savings by households [64,73], which may, in turn, affect consumption decisions related to drinking, smoking, and food expenditure. For example, as households become more cautious about the future policy environment and its impact on stability, they may reduce spending on non-essential goods, including alcohol and tobacco. Similarly, individuals may substitute away from dining out toward home-prepared meals. Evidence from the British Nutrition Foundation suggests that home-cooked meals generally contain lower levels of fat, saturated fat, and added sugars, while providing higher levels of fruits and vegetables compared to meals prepared outside the home. This substitution may, therefore, contribute to lower risks of overweight and obesity by reducing the intake of dietary fat and sugar.

At the same time, reduced household spending may also be associated with adverse health-related behaviors. If health-related goods are normal goods, tighter perceived budget constraints may be associated with lower investment in health, such as reduced medical expenditures and decreased consumption of nutritious foods. Financial concerns may also be associated with delays in seeking medical care, which could worsen conditions that benefit from early detection and treatment. In addition, precautionary saving behavior may limit spending on recreational activities, thereby reducing opportunities for physical activity.

Consequently, the relationship between EPU and health outcomes through this channel is ambiguous.

### 3.2. Time Reallocation Channel

While Ruhm [13] emphasizes the opportunity cost of time through the lens of realized job loss, EPU may influence time allocation even in the absence of unemployment. Elevated uncertainty is often associated with a general slowdown in economic activity [71,72,73], as firms may adopt a “wait-and-see” approach to investment and production [67,68,69,70]. Although this does not necessarily reduce employment, it may lead to a lower intensity of work—such as fewer overtime hours or reduced work-related commitments—thereby easing time constraints for some individuals.

Beyond these indirect effects, EPU may also affect behavior through expectation-driven adjustments. In particular, heightened uncertainty can alter the perceived opportunity cost of time, encouraging individuals to reallocate time toward health-promoting activities, such as physical exercise or preventive healthcare, as a form of self-protection against potential future shocks [11]. Increased flexibility in time use, as well as reduced work-related time pressure, may also facilitate greater engagement in social interaction and community activities, which are important for mental health and emotional well-being. As a result, periods of heightened uncertainty may be associated with healthier lifestyle behaviors through this channel.

### 3.3. Decreased Travel and Mobility Channel

Economic policy uncertainty may also be associated with health outcomes through changes in mobility. During periods of heightened uncertainty, individuals may adopt a more precautionary and risk-averse approach to discretionary activities, leading them to postpone or cancel travel plans and avoid large gatherings [76]. This voluntary reduction in mobility may, in turn, be associated with fewer traffic-related accidents and lower exposure to crowded environments, potentially reducing the risk of infectious diseases such as influenza and other communicable illnesses.

Importantly, this mechanism differs from that associated with general economic fluctuations. As emphasized by Ruhm [13], traffic-related mortality tends to be procyclical, increasing during economic expansions as work-related activity and behaviors such as drinking and driving become more prevalent; conversely, during recessions, reductions in income, employment, and overall economic activity, which mechanically reduce mobility, are correlated with fewer accidents. In contrast, reductions in mobility under heightened EPU are more likely to reflect precautionary and expectation-driven behavior, even in the absence of contemporaneous changes in labor market conditions, indicating a distinct pathway linking EPU to external-cause mortality.

### 3.4. Increased Psychological Stress Channel

Economic policy uncertainty can act as a psychosocial stressor [9,10], as it heightens concerns about future economic conditions even in the absence of realized shocks. In this context, elevated uncertainty may increase perceived stress and anxiety, which, in turn, may be associated with unhealthy coping behaviors, such as increased alcohol consumption and tobacco use [74]. From this perspective, EPU may be associated with adverse health outcomes.

Overall, the relationship between EPU and health outcomes is theoretically ambiguous, reflecting offsetting behavioral and economic mechanisms. As such, the net association remains an empirical question.

## 4. The Association Between EPU and Mortality Rates

### 4.1. Econometric Framework for Mortality Analysis

#### 4.1.1. MIDAS Model

In traditional regression frameworks, it is generally not feasible to combine dependent and independent variables observed at different sampling frequencies. This limitation becomes more pronounced in panel data settings. However, in practice, many macroeconomic indicators and health outcomes are recorded at different frequencies. For instance, economic policy uncertainty (EPU) is typically observed at a monthly or daily frequency, whereas mortality rates are commonly available at an annual frequency.

When faced with mixed-frequency data, a common approach is to aggregate high-frequency variables to match the lower frequency. For example, Antonakakis and Gupta [2] construct an annual EPU by averaging monthly observations within each year. However, such aggregation may result in a loss of valuable high-frequency information. An alternative approach is to interpolate low-frequency data to a higher frequency, but this requires selecting an appropriate interpolation method, which can be challenging. Different interpolation techniques may yield substantially different results, and inappropriate choices may distort the underlying data.

To address these limitations, Ghysels et al. [23] propose the MIDAS approach, which allows for running a regression that combines time series data sampled at different frequencies. Consider two time series $\left\{Y_{t},t=0,1,2,\ldots ,T\right\}$ and $\left\{X_{t}^{\left(m\right)},t=0,\frac{1}{m},\frac{2}{m},\ldots ,T-\frac{1}{m},T\right\}$, which are observed at low and high frequencies, respectively. Suppose that $Y_{t}$ is observed at a low frequency (e.g., annually), referred to as the reference interval. The superscript m on $X_{t}^{\left(m\right)}$ indicates the number of high-frequency observations (e.g., monthly, with m=12) within each low-frequency period. In this setting, the low-frequency variable $Y_{t}$ can be related to lagged high-frequency observations $X_{t-k/m}^{\left(m\right)}$, where k indexes the number of high-frequency lags measured backward from the end of period t. For example, in an annual–monthly setting, if k=6, then $X_{t-6/12}^{\left(12\right)}$ corresponds to the value of X in June of year t. Notation, by the help of this example, is provided below the table.
**Notation**t=2020, m=12$Y_{t}$$Y_{2020}$$X_{t}^{\left(12\right)}$$X_{2020,Dec}^{\left(12\right)}$//$X_{t-1/12}^{\left(12\right)}$$X_{2020,Nov}^{\left(12\right)}$//⋮⋮//$X_{t-11/12}^{\left(12\right)}$$X_{2020,Jan}^{\left(12\right)}$$Y_{t-1}$$Y_{2019}$$X_{t-1}^{\left(12\right)}$$X_{2019,Dec}^{\left(12\right)}$

The basic idea of the MIDAS model is to aggregate high-frequency variables within a regression framework without requiring prior temporal aggregation. The baseline MIDAS specification is given by:

(1) $$Y_{t}=\alpha +\beta W\left(L^{1/m},\theta \right)X_{t}^{\left(m\right)}+\epsilon_{t},$$

where

$$W(L^{1/m},\theta )=\sum_{k=1}^{K}w(k;\theta )L^{(k-1)/m},$$

where w(k;θ) is the lag weights parameterized as a function of a low-dimensional parameter vector θ. $L^{1/m}$ is a lag operator such that $L^{k/m}X_{t}^{\left(m\right)}=X_{t-k/m}^{\left(m\right)}$. K denotes the maximum lag order of the high-frequency observation and may be smaller or larger than m. $\epsilon_{t}$ is the error term.

As noted by Ghysels et al. [23], the number of lagged high-frequency terms in specification (1) can be large. For simplicity, we assume that the lag length K is finite. Nevertheless, even under this assumption, the number of lagged coefficients may still be substantial. For example, if the annual outcome $Y_{t}$ depends on three years of lagged monthly observations of $X_{t}^{\left(m\right)}$, then K=36 lagged coefficients would need to be estimated. To address this parameter proliferation, MIDAS models impose a functional form on the lag weights w(k;θ), allowing a large number of lagged terms to be summarized through a parametric weighting function with a small number of parameters. As a result, the slope coefficient β measures the effect of the weighted aggregation of lagged high-frequency observations $X_{t}^{\left(m\right)}$ on $Y_{t}$. Next, we consider two common parameterizations of the lag weighting function w(k;θ), each defined by a two-dimensional parameter vector $\theta =(\theta_{1};\theta_{2})$. The first one is known as the “Exponential Almon Lag”, defined as

(2) $$w(k;\theta )=\frac{e^{\theta_{1}k+\theta_{2}k^{2}}}{\sum_{k=1}^{K}e^{\theta_{1}k+\theta_{2}k^{2}}}.$$

The second one is known as the “Beta Lag”, defined as

(3) $$w\left(k;\theta \right)=\frac{f\left(\frac{k}{K},\theta_{1};\theta_{2}\right)}{\sum_{k=1}^{K}f(\frac{k}{K},\theta_{1};\theta_{2})},$$

where

$$f(a,\theta_{1};\theta_{2})=\frac{\left(a{)}^{\theta_{1}-1}(1-a{)}^{\theta_{2}-1}\Gamma (\theta_{1}+\theta_{2}\right)}{\Gamma (\theta_{1})\Gamma (\theta_{2})},\Gamma (\theta )=\int_{0}^{\infty }e^{-x}x^{\theta -1}dx.$$

In specifications (2) and (3), the weights w(k;θ) add up to 1, i.e., $\sum_{k=1}^{K}w(k;\theta )=1$. The use of parametric weighting functions allows a potentially large number of lagged terms to be represented by a small number of parameters, thereby preserving degrees of freedom. In the subsequent analysis, we employ the MIDAS model with Beta lag weights.

#### 4.1.2. Baseline Model

In this paper, we revisit Ruhm’s [13] fixed-effects model using a panel-MIDAS model to analyze the relationship between macroeconomic conditions and health outcomes:

(4) $$\begin{array}{cc} M_{st}= & \beta \sum_{k=1}^{K_{m}}w(k;\theta_{E})E_{st-(k-1)/12}^{\left(12\right)}+\lambda \sum_{k=1}^{K_{m}}w(k;\theta_{U})U_{st-(k-1)/12}^{\left(12\right)}\\ & +\eta \sum_{k=1}^{K_{q}}w(k;\theta_{PI})PI_{t-(k-1)/4}^{\left(4\right)}+\rho X_{st}+\alpha_{s}+\gamma_{t}+\epsilon_{st}, \end{array}$$

where $M_{st}$ is the mortality rate in state s at time t, E is the EPU, U is the unemployment rate, PI is the personal income per capita, X is a vector of demographic characteristics such as age, gender, race, education, and Hispanic origin. State-specific fixed effects are represented by $\alpha_{s}$, and time-period fixed effects are represented by $\gamma_{t}$. $K_{m}$ refers to the lag order of EPU and unemployment rates, while $K_{q}$ refers to the lag order of personal income per capita. The dataset consists of data at three different frequencies, as shown in the table below.
**Frequency****Variable**AnnualMortality rates, Demographic characteristicsQuarterlyPersonal income per capita (m=4)MonthlyEPU (m=12), Unemployment rate (m=12)

Since EPU may be associated with mortality over multiple periods rather than contemporaneously, we allow for distributed lag effects by including up to four years of lagged high-frequency observations for monthly EPU, monthly unemployment, and quarterly personal income per capita. This corresponds to lag orders for $K_{m}$ and $K_{q}$ ranging from 12 to 48 and from 4 to 16, respectively. For parsimony, we assume that EPU and unemployment rates share the same lag orders. This specification reflects the possibility that EPU may be associated with mortality through gradual adjustment processes—such as behavioral responses, prolonged stress exposure, labor market dynamics, and changes in healthcare utilization—that unfold over time rather than contemporaneously. Within this framework, the MIDAS coefficient captures the weighted contribution of current and past high-frequency realizations of EPU. Accordingly, it should be interpreted as the association between mortality and a distributed exposure to EPU over time, rather than the effect of a one-period change in contemporaneous EPU. In other words, the estimated coefficient reflects the cumulative impact of EPU across multiple periods, as determined by the lag weighting structure. The optimal lag length is selected using the Akaike Information Criterion (AIC). In addition, we assess the sensitivity of the results to alternative lag horizons (2-year and 6-year windows) in the robustness analysis.

To ensure valid statistical inference, we conduct a series of diagnostic tests to assess potential heteroskedasticity, serial correlation, and multicollinearity within the regression framework of model (4). For most mortality outcomes, the Modified Wald test consistently rejects the null hypothesis of homoskedasticity, indicating the presence of groupwise heteroskedasticity. In addition, the Wooldridge test rejects the null hypothesis of no first-order autocorrelation, suggesting serial correlation in the panel data. To address these issues, we report standard errors clustered at the state level for all models [77], which are robust to both heteroskedasticity and within-state serial correlation. We further assess multicollinearity using variance inflation factors (VIFs). The results indicate that multicollinearity is not a concern, as all VIF values fall below commonly accepted thresholds (e.g., mean VIF = 3.06 for total mortality). For brevity, detailed diagnostic test results for total mortality are reported in Appendix A Table A1.

To benchmark the MIDAS estimates, we conduct an OLS analysis in which high-frequency variables are aggregated into annual counterparts using equal weights, with the lag horizon set to match that selected in the MIDAS specification (4). This alignment ensures a direct comparison between the MIDAS estimates and a conventional equal-weight aggregation approach.

Moreover, following Huang et al. [11], we estimate traditional distributed lag (DL) models. In this framework, high-frequency variables are first aggregated into their annual series by averaging their monthly or quarterly values within each year, and the resulting annual series are then included with up to three annual lags. This specification provides a standard benchmark for comparison with the MIDAS approach. For brevity, we only report the results for total mortality rates in Appendix A Table A2.

#### 4.1.3. Oster Bound Test

While model (4) fully controls for economic cycle fluctuations, economic performance, demographic characteristics, and time-invariant heterogeneity, potential identification concerns remain due to omitted variables that may be correlated with both EPU and mortality. Such omitted variable bias may lead to biased estimates and affect inference. Following Ruhm [78], we perform the Oster bound test [79] to assess the sensitivity of the estimated EPU coefficient in model (4) to potential omitted variable bias.

The Oster bound approach relies on comparing coefficient estimates and R-squared values across regressions with different sets of controls, typically contrasting an uncontrolled specification with a controlled specification that includes observable covariates. However, this comparison is not directly transferable to the MIDAS context. In particular, the aggregation of high-frequency variables (e.g., EPU) depends on lag structures and weighting schemes, which may differ across specifications. As a result, the constructed regressor is not directly comparable between controlled and uncontrolled regressions, complicating the application of the standard Oster procedure.

To address these challenges, we approximate the MIDAS specification using an annualized representation. Specifically, we consider the following model:

(5) $$M=\beta \bar{E}+W_{1}+W_{2}+\epsilon ,$$

where M is the aforementioned mortality rate. $\bar{E}$ is the annual EPU, aggregated from its monthly counterparts using the lag order and weighting schemes specified in the MIDAS model (4). $W_{1}=\psi Z$ is a combination of observable control variables Z, which includes $\bar{U}$, $\bar{PI}$, demographic characteristics, state-specific fixed effects, and time-period fixed effects, and their corresponding coefficients, ψ. $\bar{U}$ and $\bar{PI}$ represent the annual unemployment rate and annual personal income, respectively. These variables are likewise aggregated from their monthly (for unemployment rates) and quarterly (for personal income) counterparts using the lag order and weighting schemes derived from model (4). $W_{2}$ is a vector of additional controls that are unobserved. Following Oster [79], we denote the EPU coefficient from the uncontrolled regression of M on $\bar{E}$ as $\overset{o}{\beta }$ with corresponding $\overset{o}{R}$, while the EPU coefficient from the controlled regression of M on $\bar{E}$ and Z as $\tilde{\beta }$ with corresponding $\tilde{R}$.

The Oster analysis involves two key parameters that capture the effect of omitted variables on empirical conclusions. The first parameter, δ, measures the extent to which selection on observable factors can provide insights into the selection on unobservable factors, particularly in explaining the variation in the treatment variable ($\bar{E}$). The parameter δ is given by

$$\delta \frac{\sigma_{1E}}{\sigma_{1}^{2}}=\frac{\sigma_{2E}}{\sigma_{2}^{2}},$$

where $\sigma_{iE}=Cov(W_{i},\bar{E})$ and $\sigma_{i}^{2}=Var(W_{i})$ with i∈{1,2}. Specifically, δ=1 suggests that observed and unobserved controls are equally important in influencing the coefficient of interest (β). When δ>1, it suggests that unobservables would have to be substantially more important than the observables and in the same direction to alter the coefficient of interest. Conversely, 0<δ<1 indicates that unobservables are less important than observables, while δ=0 implies no role of unobservables (the controlled regression). The second parameter, $R_{max}$, is the maximum R-squared from a complete regression (5). It measures the degree to which selection on observable factors can provide insights into the selection on unobservable factors, particularly in explaining variation in the outcome variable (M). The value of $R_{max}$ lies between the R-squared from the controlled regression ($\tilde{R}$) and 1.

The Oster bounds approach serves a dual purpose. It not only identifies the value of δ required for the coefficient of interest (β) to be reduced to zero, but also allows for the calculation of adjusted estimates of β for alternative assumptions about δ and $R_{max}$. The bias-adjusted estimate, denoted by $\beta^{*}$, accounts for potential omitted variable bias and is given by:

(6) $$\beta^{*}\approx \tilde{\beta }-\delta [\overset{o}{\beta }-\tilde{\beta }]\frac{R_{max}-\tilde{R}}{\tilde{R}-\overset{o}{R}},$$

where δ and $R_{max}$ are not directly observable and must therefore be specified.

Following Oster [79] and Altonji et al. [80], δ=1 is commonly used as an upper bound. The lower bound corresponds to $R_{max}=\tilde{R}$ or δ=0, which yields the controlled estimate $\tilde{\beta }$. The bounding set for β is therefore

$${∆}_{s}=[\tilde{\beta },\beta^{*}(R_{max},\delta =1)],$$

where ${∆}_{s}$ represents the range within which the true treatment effect is likely to lie when accounting for potential omitted variable bias.

Regarding the choice of $R_{max}$, Oster [79] considers $R_{max}=1$ as an upper bound and suggests parametrizing it as a function of $\tilde{R}$ i.e., $R_{max}=min(\Pi \tilde{R},1)$, with varying values of Π. Based on a sample of randomized results, Oster [79] further recommends Π=1.3 as a reasonable approximation. In our setting, however, this parameterization yields $R_{max}=1$ for all mortality outcomes. Alternatively, Black et al. [81] and Nunn and Wantchekon [82] propose $R_{max}=\tilde{R}+(\tilde{R}-\overset{o}{R})$, which implies that observed and unobserved controls are equally important in explaining the outcome. In our data, this alternative often implies values of $R_{max}$ exceeding 1, which is not feasible. To address these issues, we adopt a more conservative specification, $R_{max}=min(1.05\tilde{R},1)$, which ensures that $R_{max}$ remains within admissible bounds while avoiding corner solutions in some mortality specifications. In the baseline specification, we set δ=1 and $R_{max}=min(1.05\tilde{R},1)$. As these choices are somewhat arbitrary, we conduct additional sensitivity analyses. Specifically, we consider an alternative value of δ=0.5, following Ruhm [78], and a range of plausible values for $R_{max}\in \{0.985,0.99,0.995,1\}$ for total mortality. The results, reported in Appendix A Table A3, also indicate that the estimated EPU coefficient is not highly sensitive to omitted variable bias.

As outlined by Oster [79], this paper adopts three criteria to assess the robustness of the estimated EPU coefficient to omitted variable bias. First, the exclusion of zero from the bounding set indicates that omitted variable bias is unlikely to be a major concern. Second, $\beta^{*}$ lies within the 95% confidence interval of $\tilde{\beta }$, suggesting that the estimate from the controlled regression is similar to the bias-adjusted estimate. Third, δ>1, indicating that unobservables would need to exert a stronger influence than observables to drive the treatment effect to zero.

In this paper, we examine the robustness of the coefficient $\tilde{\beta }$ from the controlled model to assess the reliability of the estimated EPU coefficient in the MIDAS model (4). One reason to favor this is that both the MIDAS model and the controlled model incorporate the same set of control variables, ensuring consistency in accounting for potential confounders. Second, the computation of both the bias-adjusted effect $\beta^{*}$ and the parameter δ necessitates the R-squared value and the estimated EPU coefficient from the controlled model. Notably, the R-squared value from the controlled model is the same as that from the MIDAS model, and the EPU coefficients and their corresponding clustered standard errors derived from these two models exhibit only slight differences. For example, the EPU coefficient for total mortality rates in the MIDAS model is −0.0371 and 0.0187, respectively, while the corresponding values in the controlled regression are −0.0371 and 0.0210. Therefore, by ensuring that $\tilde{\beta }$ is robust to omitted variable bias, we can have greater confidence that the EPU coefficient in the MIDAS model is also robust.

### 4.2. Data for Mortality Analysis

#### 4.2.1. Mortality Rates

Annual mortality data are collected from the Centers for Disease Control and Prevention (CDC). This dataset provides state-level information, including age-adjusted total mortality rate, age-adjusted sex-specific mortality rate, and crude death rates for specific age groups and causes per 100,000 inhabitants. Using the CDC mortality data, we construct our dependent variables, namely age-adjusted total mortality rates, age-adjusted sex-specific mortality rates, crude death rates of four age groups: 15–34, 35–54, 55–64, and ≥65-years-olds, and crude death rates for nine specific causes: respiratory disease, cardiovascular disease, ischemic heart disease, cerebrovascular disease, cancer, suicide, homicide, accident, and pneumonia and influenza.

#### 4.2.2. Economic Policy Uncertainty

The US state-level EPU index is constructed by Baker et al. [83] based on newspaper coverage frequency and is measured at a monthly frequency. The state-level EPU measure is derived from information contained in sets of daily and weekly newspapers in each state, excluding national newspapers published within the state. To build the index, Baker et al. [83] use newspaper archives to search for terms related to the economy, uncertainty, and policy. For each state, they construct three separate EPU indices based on variations in the policy-related term sets.

The first index measures uncertainty arising from national policy-related sources (EPU_national), the second captures uncertainty related to state and local policy issues (EPU_state), and the third reflects uncertainty associated with both national and state policy issues (EPU_composite). In this paper, we use EPU_composite as a proxy for economic policy uncertainty. These data are available at www.policyuncertainty.com.

#### 4.2.3. Explanatory Variables

Our analysis in model (4) also includes monthly unemployment rates and quarterly personal income per capita as control variables, which are collected from the Federal Reserve Economic Data (FRED). State-year-level demographic controls are drawn from two sources. Data on population shares by age, gender, race, and Hispanic origin are obtained from the CDC, while information on educational attainment shares is obtained from the American Community Survey (ACS). Specifically, model (4) controls for the shares of the state population that are female; American Indian or Alaska Native; Black or African American; Hispanic or Latino; and in the following age groups: 15–54, 55–64, and ≥85 years. It also includes the share of the population aged 18 years and older with a bachelor’s degree. The sample covers the 50 US states and the District of Columbia over the period 2009–2020. Summary statistics for the state-level variables used in model (4) are presented in Table 1.

### 4.3. Empirical Results for Mortality Analysis

#### 4.3.1. Total Mortality Rates

Table 2 reports the estimation results for models in which the dependent variable is the age-adjusted total mortality rate. All specifications include state and year fixed effects, demographic controls, and EPU; some specifications additionally control for the unemployment rate and personal income per capita.

In the top panel, we present estimation results using the MIDAS model, in which a sufficiently long distributed lag structure of the high-frequency variables is incorporated into the specification. Across specifications, the estimated EPU coefficient is negative and statistically significant at the 5% level, indicating an inverse association between EPU and total mortality.

In the baseline specification (column a), the optimal lag structure selected by the AIC implies that mortality is associated with distributed exposure to EPU over approximately 38 months. Accordingly, the estimated coefficient reflects the association between mortality and the weighted contribution of current and past realizations of EPU over this period, rather than the effect of a contemporaneous one-period increase in EPU. Specifically, the baseline estimate indicates that a one-point increase in the weighted EPU exposure captured by the MIDAS structure is associated with a reduction of 0.0372 deaths per 100,000 population. The estimated association remains stable across alternative specifications. Excluding personal income per capita (column b) or the unemployment rate (column c) yields coefficients of −0.0346 and −0.0521, respectively.

While the absolute magnitude of −0.0372 appears small, its practical significance is better interpreted through the lens of elasticity. Evaluated at the sample means (182.8 for EPU and 762.3 for mortality; see Table 1), the estimated elasticity is:

$$Elasticity=\beta \times \left(\frac{Mean\;EPU}{Mean\;Mortality}\right)=-0.0372\times \left(\frac{182.8}{762.3}\right)\approx -0.0089.$$

This suggests that a 1% increase in EPU is associated with about a 0.009% decrease in mortality. Although small, this magnitude is comparable to existing evidence. For example, Huang et al. [11], using annual EPU and mortality in a cross-country setting, report a cumulative coefficient of approximately −0.013 based on a DL model with a two-year lag structure, which is of a similar order of magnitude.

To further illustrate the magnitude in more intuitive terms, a one-standard-deviation increase in EPU is associated with a reduction of approximately 5.9 deaths per 100,000 population (−0.0372×159.7≈−5.94), which corresponds to about 0.8% of the mean mortality rate. Although modest in relative terms, this magnitude may still be relevant at the population level. For scale, applying this estimate to a population the size of the US (about 330 million) would correspond to on the order of 19,000 fewer deaths annually, although this calculation is intended as an illustrative approximation rather than a precise national estimate. Moreover, this estimate reflects the cumulative association of EPU over time due to the distributed lag structure of the MIDAS model.

Consistent with Ruhm [13], unemployment is inversely correlated with mortality. In column (a), a one-point increase in the unemployment rate is associated with a decrease in total mortality of approximately 2.3735 per 100,000 population. This relationship remains negative but becomes smaller and statistically insignificant when personal income per capita is excluded (column b), with a reduction of 1.3912 per 100,000 population. Personal income per capita is also negatively correlated with mortality—a one-point increase in personal income per capita is associated with a reduction in mortality of approximately 3 to 4 per 100,000 population.

Importantly, the roles of unemployment and personal income per capita require careful interpretation. While these variables are commonly included as controls in prior mortality studies (e.g., Kawachi et al. [9]; Huang et al. [11]; Ruhm [13]), they may also reflect broader economic conditions that co-move with both EPU and mortality. In this sense, they could plausibly lie along pathways linking economic conditions and health outcomes. As a result, including these variables may absorb part of the variation shared with EPU, potentially attenuating the estimated association. Notably, excluding both variables (column d) yields a larger EPU coefficient (−0.0627), suggesting that the observed inverse association between EPU and mortality is not driven by their inclusion. However, the present analysis does not allow us to distinguish whether these variables primarily act as confounders or reflect pathway mechanisms.

In the bottom panel, we report estimation results from a conventional OLS specification, in which high-frequency variables are aggregated into annual counterparts by averaging over the same lag horizon selected in the MIDAS model. The results show that the estimated coefficients on EPU are negative across specifications; however, they are not statistically significant at the 5% level. Consistent with these findings, the estimated cumulative effect of EPU in the DL model is also negative but statistically insignificant across specifications (see Appendix A Table A2). Relative to the MIDAS estimates, the OLS specification is associated with weaker statistical significance of the EPU coefficients and generally larger clustered standard errors. Regarding model fit, the high $R^{2}$ values observed across specifications largely reflect the inclusion of state and year fixed effects, which capture a substantial share of the variation in mortality. Therefore, differences in $R^{2}$ should not be interpreted as evidence of superior model performance. Instead, the comparison between MIDAS and OLS is more appropriately based on coefficient stability and estimation precision. In this respect, the MIDAS framework yields more precise estimates than the simple equal-weight aggregation approach.

Table 3 reports the results of the Oster bounds analysis for the estimated association between EPU and mortality outcomes. For reference, the first row restates the EPU coefficient from the MIDAS specification (4). The second row presents the Oster bounding set, and the third row reports the bias-adjusted estimates ($\beta^{*}$) with bootstrapped standard errors in brackets, under the assumption that $R_{max}=min(1.05\tilde{R},1)$ and δ=1. The value of δ with β=0 and $R_{max}=min(1.05\tilde{R},1)$ is provided in the bottom row.

Focusing on the Oster test results for total mortality rates, we find that the Oster bounding set excludes zero, and the bias-adjusted estimates ($\beta^{*})$ fall within ±1.96 standard errors of the controlled estimates $\left(\tilde{\beta }\right)$. In particular, under the maintained assumptions, the bias-adjusted EPU coefficient is larger in magnitude than the corresponding estimate from the MIDAS model (4), yielding a statistically significant coefficient of −0.0502. The value of δ is about −3.6, suggesting that unobserved factors would need to be more than three times as influential as the observed controls and exhibit selection in the opposite direction to fully attenuate the estimated association to zero. Taken together, and given the inclusion of a rich set of controls and fixed effects in the baseline specification, these results suggest that the estimated association between EPU and mortality is not highly sensitive to omitted variable bias, although such bias cannot be entirely ruled out.

#### 4.3.2. Sex-Specific Mortality Rates

Next, we examine whether the association between EPU and mortality differs by sex. The top panel of Table 4 reports the results of this analysis based on two alternative specifications. All models include state and year fixed effects as well as demographic controls, while column (2) additionally controls for unemployment rates and personal income per capita.

In column (1), the estimated association between EPU and mortality is larger in magnitude for males than for females. Specifically, a one-point increase in EPU is associated with a decrease of 0.0626 deaths per 100,000 population for males and 0.0507 for females, indicating that the estimated association for females is approximately 19% smaller than that for males.

When unemployment and personal income per capita are included (column 2), the estimated EPU coefficients remain negative and statistically significant, although their magnitudes are attenuated by approximately 20% for both groups. Similarly to the total mortality results, this attenuation suggests that unemployment and income may partly capture broader macroeconomic conditions associated with both EPU and mortality. Consistent with the results for total mortality, unemployment and personal income per capita are negatively correlated with mortality, with somewhat larger magnitudes observed for males. For example, a one-point increase in the unemployment rate is associated with a reduction of 2.3308 deaths per 100,000 population for males, compared with 1.9635 for females. Similarly, a one-point increase in personal income per capita is associated with increases of 4.4128 and 3.0736 deaths per 100,000 population for males and females, respectively.

The Oster bounds results (Table 3) provide additional evidence on the sensitivity of these estimates. Across sex-specific mortality outcomes, the bounding sets exclude zero, and the bias-adjusted estimates ($\beta^{*}$) remain within the 95% confidence interval of the controlled estimates. In addition, the implied values of δ exceed one in absolute value, suggesting that unobserved factors would need to be substantially more influential than observed controls to fully attenuate the estimated associations to zero. The bias-adjusted coefficients are also somewhat larger in magnitude, particularly for males, which is consistent with the patterns observed in Table 4.

#### 4.3.3. Age-Specific Mortality Rates

The remainder of Table 4 reports results for age-specific mortality across four groups: 15–34, 35–54, 55–64, and ≥65 years. Column (1) shows that the estimated association between EPU and mortality is negative across all age groups, although statistical significance varies, with weaker evidence for the 55–64 group (significant at the 10% level). The magnitude of the estimated association appears to increase with age. Specifically, a one-point increase in EPU is associated with a reduction in mortality of 0.3184 per 100,000 population for those aged ≥65, compared with 0.0158, 0.0510, and 0.1022 for the 15–34, 35–54, and 55–64 cohorts, respectively. This finding is consistent with Huang et al. [11], who also note that the health-improving associations of EPU are particularly pronounced among the elderly.

One plausible explanation for the stronger association between EPU and mortality among individuals aged ≥65 is that EPU operates primarily through expectations, which may have a disproportionate impact on older populations. Compared to younger individuals, older adults rely more heavily on fixed incomes, savings, and pensions, making them more sensitive to uncertainty about future economic conditions, healthcare costs, and financial security. As a result, heightened uncertainty may induce stronger precautionary responses, such as reducing exposure to non-essential activities or adopting more risk-averse behaviors (see Section 3), which could lower exposure to external health risks. In addition, older individuals are generally more vulnerable to short-term changes in environmental and behavioral factors, so even modest adjustments in daily activities may translate into relatively larger health benefits. Taken together, these expectation-driven and behavioral responses may be more pronounced among the elderly, potentially contributing to the stronger association between EPU and mortality observed in this group, although this interpretation remains suggestive.

Several points emerging from the results in column (2) are noteworthy. Firstly, the inclusion of unemployment rates and personal income does not substantially alter the estimated EPU coefficients for cohorts aged 35 and above. In contrast, the estimated EPU coefficient for the 15–34 group declines by approximately 48% when unemployment and personal income are included. This pattern suggests that labor market conditions—proxied by unemployment and income—may capture part of the macroeconomic variation associated with both EPU and mortality that is particularly relevant for younger individuals. Given that younger cohorts are generally more directly exposed to labor market fluctuations, these controls may absorb overlapping variation related to both EPU and mortality, leading to a larger attenuation of the estimated EPU coefficient.

Secondly, in accordance with Ruhm [13], mortality rates are generally inversely correlated with unemployment, although this association is statistically significant only for the 15–34 and ≥65 cohorts. The magnitude of this association is largest for individuals aged ≥65, for whom a one-point increase in unemployment is associated with a reduction of 10.3572 deaths per 100,000 population. This pattern is consistent with prior studies that suggest that procyclical mortality in the US is more pronounced among older populations [15,84], who are less directly involved in the labor market. For example, Stevens et al. [84] point out that the majority of additional deaths during periods of economic growth occur among the elderly, accounting for 75% of the roughly 6700 additional deaths (across all ages) predicted from a one-point drop in the unemployment rate.

Lastly, personal income per capita is negatively associated with mortality across all groups, although the estimates are not statistically significant for the 35–54 cohort at the 5% level. The magnitude of the association is larger for older groups (−8.4661 for ages 55–64 and −12.9484 for ≥65). Notably, for the 55–64 cohort, income appears more strongly correlated with mortality than EPU or unemployment, as the latter variables exhibit only marginal or insignificant associations in the full specification.

Additionally, the results in Table 3 for age-specific mortality satisfy the Oster robustness criteria, suggesting that the estimated associations are not highly sensitive to selection on unobservables under the maintained assumptions.

#### 4.3.4. Cause-Specific Morality Rates

Table 5 summarizes the results of the subgroup analysis by cause-specific mortality. The categories include deaths attributed to respiratory disease, cardiovascular disease, ischemic heart disease, cerebrovascular disease, cancer, suicide, homicide, accidents, and pneumonia and influenza. The estimates are based on two specifications that include state and year fixed effects, demographic variables, and EPU, with and without additional controls for unemployment rates and personal income per capita.

When unemployment and personal income per capita are not included (column 1), EPU is positively associated with mortality from cerebrovascular disease, cancer, and suicide. However, the statistical evidence for cerebrovascular disease and cancer is relatively weak, as these estimates are only significant at the 10% level. In contrast, the positive association with suicide is statistically significant and consistent with prior studies (e.g., Antonakakis and Gupta [2]; Huang et al. [11]). Specifically, a one-point increase in EPU is associated with an increase of 0.0028 deaths per 100,000 population from suicide. Conversely, EPU is significantly negatively associated with mortality from homicide, accidents, and pneumonia and influenza, with the largest magnitude observed for accident-related mortality. A one-point increase in EPU is associated with a reduction of 0.0094 deaths per 100,000 population from accidents, compared to reductions of 0.0019 and 0.0046 for homicide and pneumonia and influenza, respectively. For deaths from respiratory disease, cardiovascular disease, and ischemic heart disease, the estimated associations with EPU are not statistically significant at conventional levels.

Controlling for the unemployment rate and personal income per capita (column 2) introduces some changes in these estimated associations. In particular, the coefficients for respiratory disease and ischemic heart disease become statistically significant at the 10% level, with point estimates of 0.0051 and 0.0117, respectively. However, the evidence for ischemic heart disease remains relatively weak given that it is only marginally significant. In contrast, the association for cerebrovascular disease, which is marginally significant in column (1), becomes statistically insignificant. Furthermore, the associations between EPU and mortality from suicide, homicide, accidents, and pneumonia and influenza persist after controlling for unemployment and income. Although the estimated coefficients are slightly attenuated, the changes are modest and do not materially alter the overall pattern of results, suggesting that these associations are relatively stable to the inclusion of income and labor market variables.

Interestingly, the results from both specifications (columns 1 and 2) indicate that EPU is not statistically significantly associated with mortality from chronic diseases—namely respiratory disease, cardiovascular disease, ischemic heart disease, cerebrovascular disease, and cancer—at the 5% significance level, with the exception of respiratory disease in column (2). As such, the evidence for chronic disease outcomes remains limited. One possible explanation for these findings relates to the latent nature of chronic disease progression. Unlike external causes of mortality, the physiological effects of prolonged stress or lifestyle changes on chronic conditions typically unfold over a longer time horizon. While our empirical framework incorporates lagged effects of EPU for up to four years, this finite window may be insufficient to fully capture the gradual accumulation of health risks associated with policy-related uncertainty. As a result, the estimated associations for these outcomes may appear weaker and less precisely estimated. In contrast, the relationship between EPU and external causes is heterogeneous: while mortality from homicide and accidents declines with higher EPU, suicide shows a positive association, indicating that external causes are not uniformly affected and may operate through different mechanisms.

The divergent associations between EPU and chronic disease mortality and external causes of death highlight the complex ways in which policy-related economic uncertainty is related to health outcomes. To further explore potential mechanisms, the subsequent sections examine how lifestyle behaviors are associated with changes in EPU. Understanding these behavioral patterns may provide insights for the design of public health strategies aimed at reducing mortality.

## 5. The Association Between EPU and Lifestyle Behaviors

### 5.1. Econometric Framework for Lifestyle Analysis

We next use US microdata to examine the association between EPU and lifestyle-related outcomes. This complementary analysis aims to assess whether changes in individual behaviors are consistent with the patterns observed for mortality. The basic empirical specification is as follows:

(7) $$\begin{array}{cc} H_{ist}= & \beta E_{st}+\lambda U_{st}+\rho X_{ist}+\alpha_{s}+\gamma_{t}+\epsilon_{ist}, \end{array}$$

where $H_{ist}$ represents lifestyle behavior indicators for individual i in state s interviewed in year-month t (e.g., December 2020). $E_{st}$ and $U_{st}$ represent EPU and unemployment rates in state s at time t, respectively. X is a vector of individual-level demographic characteristics, including income, age, gender, race, education, marital status, and Hispanic origin. State-specific fixed effects are represented by $\alpha_{s}$, and time-period fixed effects by $\gamma_{t}$. Standard errors are clustered at the state level.

In this study, lifestyle behavior indicators include both health-related behaviors and time allocation across various activities, as described in the following section.

### 5.2. Data for Lifestyle Behavior Analysis

#### 5.2.1. Health-Related Behaviors

Microdata on the prevalence of risky health behaviors are obtained from the Behavioral Risk Factor Surveillance System (BRFSS), which is conducted by the CDC. The BRFSS collects monthly, state-level data on adults’ health-related risk behaviors, preventive health practices, and chronic health conditions.

The outcomes studied include the following: alcohol and cigarette use, physical activity, and Body Mass Index (BMI). Alcohol consumption is analyzed using two dichotomous variables. The first indicates current drinking status, defined as having consumed at least one alcoholic beverage in the past 30 days. The second identifies binge drinking, defined using gender-specific thresholds: five or more drinks for men and four or more drinks for women on at least one occasion during the past 30 days. Smoking status follows the BRFSS definition. A binary indicator is constructed for current smoking based on responses to the questions: “Have you smoked at least 100 cigarettes in your entire life?” and “Do you now smoke cigarettes every day, some days, or not at all?” Respondents who answer “yes” to the first question and “every day” or “some days” to the second are classified as current smokers. Physical activity is measured using a BRFSS core question: “During the past month, other than your regular job, did you participate in any physical activities or exercises such as running, calisthenics, golf, gardening, or walking for exercise?” A binary indicator of physical inactivity is defined as responding “no” to this question. BMI-based measures are used to construct indicators for overweight and obesity. Overweight is defined as a BMI of 25.00 to 29.9, and obesity as a BMI of 30.0 to 99.8. Respondents with missing, “don’t know,” or refused responses are excluded from the analysis.

In addition, the BRFSS includes a “Healthy Days Module” that measures health-related quality of life. This module assesses individuals’ self-reported general health, physical health, mental health, and activity limitations through four questions. The first question asks, “In general, would you say your health is poor, fair, good, very good, or excellent?” Based on these responses, we construct a five-level ordinal variable, where 1 denotes excellent health and 5 denotes poor health. The second and third questions report the number of days in the past 30 days during which the respondent’s physical or mental health was not good. Respondents reporting at least one such day are further asked about the number of days in the past 30 days in which poor physical or mental health limited their usual activities. We construct indicator variables for frequent physical distress, frequent mental distress, and frequent activity limitation, defined as reporting 14 or more days in each respective category. Respondents with missing, “don’t know,” or refused responses are excluded from the analysis.

#### 5.2.2. Time Allocation

Data on time allocation across activities are collected from the American Time Use Survey (ATUS). The ATUS is an annual survey conducted by the US Bureau of Labor Statistics (BLS) that records how individuals allocate their time over a 24-h period, including activities such as socializing, travel, household production, and sports. The ATUS sample is drawn from households that have completed their final interview in the Current Population Survey (CPS). From each eligible household, one respondent aged 15 or older is selected for a time-use interview conducted two to five months after the final CPS interview. This design yields a nationally representative sample of the U.S. population. The ATUS does not typically include state identifiers in the public-use files. To obtain state-level information, we link ATUS respondents to their corresponding CPS records by survey year. This linkage allows us to assign the state of residence and analyze geographic variation in time allocation.

According to the conceptual model in Section 3, our analysis focuses on four specific outcomes: time (in minutes) spent on traveling, socializing, sports, and personal medical care. Following Aguiar et al. [85], the socializing category includes activities such as socializing and communicating with others, attending or hosting social events, playing games, and making telephone calls. We additionally include volunteer activities within this category. Personal medical care comprises time spent on health-related self-care as well as the use of medical and care services. For each outcome, we account for both active time and time spent waiting.

#### 5.2.3. Individual Characteristics

Demographic information, including sex, race, age, ethnicity, educational attainment, marital status, and income level, is available for each respondent in both the BRFSS and ATUS datasets. Following Ruhm [13], the individual-level demographic characteristics included in model (7) comprise household income levels, years of age, and indicator variables for college graduation or higher, Hispanic origin, female, married status, American Indian or Alaska Native, and Black or African American. The sample covers the 50 US states and the District of Columbia over the period 2009–2020. Summary statistics for the individual-level variables used in model (7) are reported in Table 6 and Table 7 for the BRFSS and ATUS samples, respectively.

### 5.3. Empirical Results for Lifestyle Behavior Analysis

Table 8 reports the marginal effects of EPU on risky health behaviors from model (7). For ease of interpretation, the results of probit models are interpreted as percentage changes in risky health behaviors associated with a one percent change in EPU, which is obtained by multiplying the estimated EPU coefficient shown in Table 8 by the product of the weighted mean of EPU and one percent, and then multiplying the result by 100%. For example, the estimated coefficient for current drinking (−0.000035) implies that a one percent increase in EPU, evaluated at a baseline level of 188.7, is associated with a 0.0066% lower probability of being a current drinker ((−0.000035×188.7×1)×100).

Our analysis uncovers an inverse relationship between EPU and risky health behaviors related to alcohol and tobacco consumption. Specifically, a one percent increase in EPU is associated with reductions in the predicted probability of being a current drinker, binge drinker, and current smoker by 0.0066%, 0.0028%, and 0.0066%, respectively, although the estimate for binge drinking is not statistically significant. According to Section 3, this may imply that although some individuals might turn to alcohol and tobacco to cope with increased psychological stress, precautionary behavioral shifts appear to be the dominant force. Specifically, the tendency to reduce discretionary spending in the face of uncertainty may lead to a net decrease in the consumption of these health-harming goods. Given that smoking and alcohol abuse are leading risks for preventable death in the US, the observed associations with lower prevalence of current drinking and smoking may be relevant for understanding broader population health patterns.

EPU is not significantly associated with the prevalence of physical inactivity. This finding is consistent with the presence of competing effects operating through the “Decreased Household Spending” and “Time Reallocation” channels discussed in Section 3. On the one hand, increased uncertainty may induce precautionary saving and tighter perceived budget constraints, which could limit spending on exercise-related goods and services. On the other hand, heightened uncertainty may be associated with shifts in time allocation toward health-promoting activities such as physical exercise. As a result, these opposing forces may offset each other, leading to a statistically insignificant association between EPU and physical inactivity. Consistent with this interpretation, our results on time allocation to sports further indicate that time spent on sports activities is largely unresponsive to changes in EPU.

Overweight and obesity together are the second leading cause of preventable death in the US. Our BMI results indicate a negative association between EPU and body weight, with a coefficient of −0.0204. The dichotomous outcomes further support this pattern. Specifically, a one percent increase in EPU is associated with reductions in the predicted probability of being overweight or obese combined and being obese by 0.0064 and 0.0062 percentage points, respectively. As outlined in Section 3, one possible interpretation is that increased uncertainty may induce adjustments in household consumption behavior through the “Decreased Household Spending” channel, as individuals shift toward relatively less costly or more home-prepared food options, which are often lower in fat and sugar content.

Individuals are more likely to report being in better health during periods of higher EPU, as indicated by the negative EPU coefficient of −0.000016, which suggests a shift toward better self-reported health categories. In contrast, the associations between EPU and the probabilities of experiencing mental and physical distress are not statistically significant. As discussed in Section 3, the absence of a significant association with mental distress may reflect offsetting mechanisms. On the one hand, heightened uncertainty may be associated with changes in time allocation, including a modest increase in time spent on socializing, as indicated by the EPU coefficient of 0.0076 (significant at the 10% level). On the other hand, EPU may also be associated with increased psychological stress and anxiety related to future economic conditions, potentially offsetting these effects. Similarly, the lack of a significant association between EPU and physical distress may reflect limited changes in related behaviors. Consistent with this interpretation, our results also suggest that EPU is not significantly associated with physical inactivity or time allocated to personal medical care. In line with this pattern, the likelihood of experiencing activity limitations among individuals reporting recent physical or mental conditions is also not significantly associated with EPU.

There is no statistically significant relationship between time spent on personal medical care and EPU. As discussed in Section 3, this pattern may reflect offsetting effects. Heightened uncertainty may reduce the perceived opportunity cost of time and reallocate time toward preventive and health-related activities. At the same time, EPU may be associated with precautionary saving and tighter perceived budget constraints, which could lead individuals to delay or forgo medical care. As a result, the net effect of these opposing forces may be limited.

Furthermore, to assess the relationship between EPU and travel-related behavior, we examine the time allocated to tourism activities. The results indicate a negative association between EPU and time spent on travel, with a one-point increase in EPU corresponding to a reduction of 0.0138 min per day. This pattern is consistent with a precautionary and risk-averse response to heightened uncertainty, whereby individuals may reduce discretionary travel. This reduction in mobility may be relevant when interpreting the observed negative association between EPU and mortality from pneumonia and influenza.

### 5.4. Long-Term Associations

To account for the possibility that behavioral responses may evolve gradually over time, we examine longer-term associations between economic conditions and risky health behaviors, rather than focusing solely on contemporaneous relationships. This is achieved by incorporating distributed lag structures for EPU and unemployment. Specifically, we replace contemporaneous EPU and unemployment in model (7) with weighted aggregates of their lagged values, using the weighting scheme and lag orders derived from the MIDAS specification for total mortality in model (4). Consequently, model (7) becomes:

(8) $$\begin{array}{cc} H_{ist}= & \beta (\sum_{k=0}^{K-1}w(k;\theta_{E})E_{st-k})+\lambda (\sum_{k=0}^{K-1}w(k;\theta_{U})U_{st-k})+\rho X_{ist}+\alpha_{s}+\gamma_{t}+\epsilon_{ist}, \end{array}$$

where the lag length, K, and the weighting schemes, $w(k;\theta_{E})$ and $w(k;\theta_{U})$, are derived from the MIDAS model (4) for total mortality rates. All other notation is consistent with that in model (7).

The results of this analysis are displayed in Table 9. These results are broadly consistent with our preceding findings in Table 8, suggesting that higher EPU is associated with reduced prevalence rates of drinkers and smokers, improved maintenance of a healthy weight range, and enhanced self-reported health. As expected, when longer-term effects are considered, the associations between EPU and these health-related outcomes appear more pronounced than contemporaneous estimates. In particular, the association between EPU and binge drinking becomes statistically significant, with a one percent increase in EPU associated with a 0.0070% lower predicted probability of binge drinking.

## 6. Robustness Check

### 6.1. Model Specification

In this section, we conduct additional robustness checks on our baseline results for total mortality rates in Table 2.

First, given that the association between EPU and mortality may vary across states due to differences in socioeconomic conditions, healthcare infrastructure, and policy environments, we augment our baseline model (4) by including state-specific linear time trends. This specification helps account for unobserved factors that evolve differently across states over time.

Second, we truncate our sample at 2019 to avoid confounding effects from the COVID-19 pandemic. The pandemic led to unprecedented levels of economic uncertainty and had a direct impact on death rates due to the virus itself. This exclusion ensures that our analysis remains unaffected by the pandemic and its associated effects on mortality rates.

Third, to assess potential reverse causality, we implement two robustness checks. First, we re-estimate model (4) using a one-year lag of EPU to ensure that the measured uncertainty precedes the observed health outcomes. Second, we conduct a placebo test by replacing current EPU with its one-year lead. If the baseline results are driven by contemporaneous shocks or reverse feedback, the lead variable would be expected to exhibit a statistically significant association with mortality. Conversely, an insignificant coefficient on the lead variable would be consistent with the interpretation that the observed relationship is not driven by reverse causality or forward-looking bias.

Finally, diagnostic tests reported in Table A1 indicate the presence of first-order autocorrelation in the total mortality models. While the baseline specifications employ cluster-robust standard errors to address this issue, we conduct an additional robustness check. Specifically, we augment model (4) by including a lagged dependent variable to account for potential persistence in mortality. This model is estimated using a dynamic panel GMM approach.

The results of these analyses are presented in Table 10. As shown, the inclusion of state-specific time trends attenuates the estimated association between EPU and mortality (column 1). Specifically, in the baseline specification, a one-point increase in EPU is associated with a reduction of 0.0372 deaths per 100,000 population; this effect decreases to 0.0231 when state-specific time trends are included. Truncating the sample at 2019 yields an EPU coefficient of −0.0407 (column 2). This slightly larger magnitude suggests that the inclusion of the pandemic period may have attenuated the underlying inverse relationship between EPU and mortality. Excluding the COVID-19 period, therefore, reveals a somewhat stronger relationship. Column (3) shows that the lagged EPU remains negatively and statistically significantly associated with mortality, while the lead EPU in column (4) is statistically insignificant. This pattern is consistent with the interpretation that the observed relationship is not driven by reverse causality. Turning to column (5), the inclusion of a lagged dependent variable yields a positive and statistically significant coefficient on lagged mortality, while the EPU coefficient remains negative and statistically significant, with only modest changes in magnitude. Overall, these robustness analyses demonstrate that while the magnitude of the EPU coefficient varies across specifications, the negative association remains stable, reinforcing the reliability of our baseline findings.

### 6.2. Alternative Lag Polynomials

Since the weighting function is a key component of the MIDAS model, its specification may affect the estimation results. We therefore explore whether our estimation results are robust to alternative choices of the weighting function. To this end, we re-estimate model (4) using the MIDAS approach with Exponential Almon lag polynomials. The results of this analysis, presented in column (1) of Table 11, are similar to those in Table 2. The magnitude, statistical significance, and sign of the estimated EPU coefficient remain largely unchanged, indicating that our baseline results are robust to the choice of weighting function.

### 6.3. Alternative Economic Policy Uncertainty Indices

As another robustness check, we utilize two alternative EPU indices from Baker et al. [83] as proxies for economic policy uncertainty. One index captures uncertainty originating from national policy-related sources within a state (EPU_national), and the other measures uncertainty stemming from state and local policy issues (EPU_state).

These results, reported in columns (2) and (3) of Table 11, again confirm the inverse association between EPU and mortality, providing additional support for our main findings. Notably, when focusing on national policy-related economic uncertainty (EPU_national), the estimated coefficient is −0.0370, which is very similar in magnitude to the baseline estimate of −0.0372, suggesting that national-level uncertainty yields broadly comparable associations with mortality. Conversely, uncertainty originating from state and local policy issues (EPU_state) yields a larger coefficient of −0.0605, indicating a stronger estimated association relative to the baseline measure.

### 6.4. Alternative Maximum Lag Horizons

While the baseline specification allows for up to four years of lagged effects, the appropriate temporal horizon over which EPU is associated with mortality is not known a priori. To assess the sensitivity of our results to this modeling choice, we re-estimate model (4) using alternative lag windows corresponding to shorter and longer horizons. Specifically, we consider a 2-year maximum lag structure (up to 24 months for monthly variables and 8 quarters for quarterly variables) and a 6-year maximum lag structure (up to 72 months and 24 quarters, respectively). Within each specification, the optimal lag length is again selected using the AIC.

The results, reported in Table 12, indicate that the estimated associations between EPU and mortality remain qualitatively similar across alternative lag horizons. In particular, both the sign and statistical significance of the EPU coefficient are consistent with the baseline specification. While the estimated magnitudes vary modestly—from −0.0404 under the 2-year specification to −0.0454 under the 6-year specification—they remain broadly comparable to the baseline estimate of −0.0372. Although the estimated coefficient under the 2-year specification remains qualitatively similar to the baseline estimate, the selected lag length for monthly variables (EPU and unemployment) reaches the imposed upper bound in the shorter-horizon specification. This suggests that the association between EPU and mortality may extend beyond the restricted 2-year window. Accordingly, the 4-year baseline specification is retained as it provides greater flexibility to capture potentially longer-run dynamics in the relationship between EPU and mortality. This suggests that the estimated associations are not sensitive to the choice of the maximum lag horizon.

## 7. Discussion

This paper examines the relationship between EPU and mortality, with a focus on potential lifestyle-related channels, including decreased household spending, changes in time allocation, reduced travel and mobility, and increased psychological pressure. Given the presence of mixed-frequency data, we use a panel-MIDAS model for the analysis. Building on Ruhm’s [13] mortality model, we provide new evidence on the association between EPU and mortality and assess the robustness of our findings through a range of empirical tests, including the Oster bound test.

The empirical results indicate a statistically significant inverse association between EPU and total mortality rates. Specifically, a one-standard-deviation increase in EPU is associated with a reduction of approximately 5.9 deaths per 100,000 population. Although the estimated magnitude of this association is modest, it can still be meaningful from a public health perspective when applied to large populations. Moreover, the magnitude of this association is comparable to estimates reported in studies examining the association between EPU and health outcomes (e.g., [11]). The results also suggest that the MIDAS model yields more precise estimates than the OLS specification, highlighting the advantages of incorporating mixed-frequency data.

Sex-specific analysis indicates that the association between EPU and mortality is more pronounced among men than women, with male mortality rates exhibiting larger declines during periods of higher EPU. Age-specific results further suggest that older populations are more sensitive to variation in EPU. In particular, a one-point increase in EPU is associated with a reduction of 0.2826 deaths per 100,000 population among individuals aged 65 and above. This pattern suggests that older populations may be more sensitive to variation in EPU. However, the evidence for the 55–64 age group is weaker, with statistical significance observed only at the 10% level.

Notably, our examination of age-specific mortality in relation to unemployment is consistent with prior research findings. Previous studies provide strong evidence that the impact of unemployment on mortality is most pronounced among the elderly, suggesting that the direct effects of changes in labor market conditions may not fully explain these mortality patterns [15,38]. By incorporating EPU into Ruhm’s [13] mortality model, our results further support these findings.

Cause-specific mortality analysis highlights heterogeneous associations between EPU and different mortality categories. Specifically, EPU is positively associated with mortality from respiratory diseases and suicide, while it is inversely associated with mortality from homicide, accidents, and pneumonia and influenza. These heterogeneous patterns suggest that the association between EPU and mortality differs across causes of death, potentially reflecting differences in behavioral adjustments, exposure risks, and baseline vulnerability across disease categories and external causes.

Our analysis of lifestyle-related behaviors suggests that heightened EPU is significantly associated with a lower prevalence of current drinking and smoking, an increased likelihood of maintaining a healthy weight range, improved self-reported health, and reduced time spent traveling. However, it is important to note that these findings offer suggestive, rather than definitive, evidence that behavioral adjustments accompany periods of elevated policy uncertainty. Furthermore, the data do not point to a single, uniform behavioral channel. Instead, the divergent patterns observed across health behaviors—coupled with the heterogeneous results in cause-specific mortality—indicate that the relationship between EPU and population health is likely shaped by multiple mechanisms that may operate in different directions and with varying intensity across outcomes.

This paper makes contributions to three strands of literature. First, it contributes to the methodological literature examining the association between health outcomes and macroeconomic conditions. By employing the MIDAS framework, more efficient use of mixed-frequency data is achieved, allowing high-frequency information to be incorporated into models with low-frequency outcomes. To the best of our knowledge, this study is the first to use the MIDAS model to examine the association between EPU and health outcomes. Second, although the literature on business cycles and health outcomes is extensive, evidence on the relationship between economic policy uncertainty and health status remains limited. This paper contributes to this area by examining sex-, age-, and cause-specific variations in mortality associated with EPU. Third, this paper extends the literature by exploring lifestyle behaviors, including health-related risky behaviors and time allocation across various activities, as potential channels through which EPU is associated with mortality outcomes.

Despite the inclusion of state and year fixed effects, a rich set of controls, and the application of Oster bounds as a sensitivity analysis, several limitations remain. First, these approaches cannot fully eliminate concerns about residual confounding arising from unobserved time-varying factors that may be correlated with both EPU and mortality. Furthermore, while the lagged specifications and placebo tests presented in the robustness section help mitigate concerns about contemporaneous reverse relationships, the possibility of dynamic feedback between economic conditions and health outcomes cannot be entirely ruled out. Second, while the Oster bounds provide a useful sensitivity analysis for assessing the potential impact of omitted variable bias, they are not a formal identification strategy and rely on assumptions regarding the relative selection on observables and unobservables, which may not hold in all contexts. Therefore, these results should be viewed as suggestive evidence of an association between EPU and mortality under plausible assumptions about omitted variable bias, rather than causal effects. Future research could employ stronger identification strategies, such as instrumental variables or natural experiments. Finally, the use of aggregated state-level data raises concerns about ecological inference, as associations observed at the state level may not necessarily reflect individual-level relationships between economic policy uncertainty and health outcomes. To partially address this issue, we complement the aggregate analysis with individual-level microdata on lifestyle-related behaviors, allowing us to examine whether patterns at the individual level are consistent with the aggregate findings. While this combined approach provides additional context, it does not fully resolve concerns regarding cross-level inference.

## 8. Conclusions

This paper provides new evidence on the association between EPU and mortality, using a panel MIDAS framework to exploit mixed-frequency data. Our findings indicate that higher EPU is generally associated with lower total mortality, although the magnitude of this relationship is modest and varies across demographic groups. In particular, the association is most pronounced among the retirement-age group.

Cause-specific mortality analysis highlights heterogeneous patterns: EPU is inversely associated with deaths from accidents, homicide, and pneumonia/influenza, but positively associated with suicide and respiratory disease mortality, with several chronic disease outcomes showing statistically insignificant associations at the 5% significance level. Lifestyle-related analyses provide suggestive evidence that behavioral adjustments—such as reduced smoking and alcohol consumption, healthier weight maintenance, and decreased time spent traveling—may partly accompany periods of elevated policy uncertainty, although no single mechanism fully explains the observed patterns.

Overall, our results underscore that the relationship between EPU and health is complex and multifaceted, shaped by multiple, potentially offsetting mechanisms. While robust to a range of specifications, the findings remain associative, and further research using stronger identification strategies is needed to clarify causal pathways. Nonetheless, this study contributes to a better understanding of how policy-related economic uncertainty is associated with population health and highlights the importance of considering heterogeneity across outcomes, demographic groups, and behavioral channels.

## Figures and Tables

**Table 1:** Summary Statistics for State-level Variables Used in Model (4).

Variables	Mean	Std. Dev.
EPU_composite	182.8	159.7
EPU_national	122.3	102.5
EPU_state	96.6	116.8
Unemployment rate (%)	6.1	2.6
Personal income per capita	47.5	9.6
Demographic Characteristics (%)		
Female	50.6	0.8
American Indian or Alaska Native	2.2	3.3
Black or African American	14.8	11.9
Hispanic or Latino	11.4	10.1
15–54 years old	53.2	2.4
55–64 years old	12.8	1.1
≥85 years old	1.9	0.4
Bachelor’s degree or higher	30.4	6.5
Type of Mortality Rate (per 100,000 population)		
Total	762.3	92.5
Male	901.5	111.6
Female	646.0	78.3
15–34-year-olds	101.2	25.6
35–54-year-olds	320.0	72.8
55–64-year-olds	899.4	184.9
≥65-year-olds	4297.6	431.7
Respiratory disease [J20-J47, U04]	50.9	13.9
Cardiovascular disease [I00-I78]	261.5	47.3
Ischemic heart disease [I20-I25]	115.6	27.7
Cerebrovascular disease [I60-I69]	43.9	8.2
Cancer [C00-C97]	190.4	28.7
Suicide [X60-X84, Y87.0]	15.5	4.4
Homicide [X85-Y09, Y87.1, Y35, Y89.0]	5.7	3.5
Accident [V01-X59, Y85-Y86]	51.0	12.9
Pneumonia and influenza [J09-J18]	17.4	4.9

Note: Data on death rates are collected from the Centers for Disease Control and Prevention (CDC). Data on Economic Policy Uncertainty (EPU) are collected from www.policyuncertainty.com. Information on unemployment rates and personal income is collected from the Federal Reserve Economic Data. Data on population shares by age, gender, race, and Hispanic origin are collected from the CDC. Data on educational attainment are collected from the American Community Survey. ICD-10 codes for specific causes of death categories are shown in square brackets, consistent with those used by Heutel and Ruhm [38]. The sample covers the period from 2009 to 2020 and includes all 50 US states and the District of Columbia.

**Table 2:** Parameter Estimates of the Determinants of Total Mortality Rates.

**Variables**	**MIDAS Estimates**			
	**(a)**	**(b)**	**(c)**	**(d)**
EPU	−0.0372 **	−0.0346 **	−0.0521 **	−0.0627 **
	(0.0178)	(0.0168)	(0.0261)	(0.0271)
Unemployment	−2.3735 ***	−1.3912		
	(0.9529)	(0.9965)		
Personal income per capita	−3.7330 ***		−3.3964 ***	
	(0.9721)		(0.8398)	
$K_{m}$	38	15	24	24
$K_{q}$	15		14	
$R^{2}$	0.9800	0.9786	0.9798	0.9784
**Variables**	**OLS estimates**			
	**(a)**	**(b)**	**(c)**	**(d)**
EPU	−0.0438	−0.0407	−0.0514 *	−0.0589 *
	(0.0364)	(0.0256)	(0.0310)	(0.0320)
Unemployment	−2.4680 **	−1.3448		
	(1.1955)	(1.3013)		
Personal income per capita	−3.4417 ***		−2.9898 ***	
	(0.7555)		(0.6905)	
$R^{2}$	0.8308	0.8221	0.8300	0.8216

Note: *** *p* < 0.01, ** *p* < 0.05, * *p* < 0.1. The dependent variables are age-adjusted total mortality rates. All specifications include state and year fixed effects, demographic controls, and EPU. For the OLS estimates, annual counterparts for EPU, unemployment, and personal income per capita are constructed by averaging their high-frequency values over the lag horizon selected in the corresponding MIDAS model. Standard errors, clustered at the state level, are reported in parentheses.

**Table 3:** Oster Bounds Analysis across Total, Sex-, Age-, and Cause-Specific Mortality.

**Variables**	**Total**	**Male**	**Female**	**15–34-Year-Olds**
MIDAS coefficient	−0.0372 **	−0.0494 **	−0.0416 **	−0.0082 **
(clustered S.E.)	(0.0178)	(0.0214)	(0.0210)	(0.0038)
Oster bounding set $\left[\tilde{\beta },\beta^{*}\right]$	[−0.0502; −0.0372]	[−0.0716; −0.0494]	[−0.0451; −0.0416]	[−0.0148; −0.0082]
Bias-adjusted coefficient $\beta^{*}$	−0.0502 **	−0.0716 **	−0.0451 ***	−0.0148 ***
	[0.0245]	[0.0292]	[0.0156]	[0.0052]
δ statistic	−3.5688	−2.7911	−18.6799	−1.5409
**Variables**	**35–54-Year-Olds**	**55–64-Year-Olds**	**≥65-Year-Olds**	**Respiratory**
MIDAS coefficient	−0.0442 **	−0.0902 *	−0.2826 ***	0.0051 **
(clustered S.E.)	(0.0216)	(0.0486)	(0.0875)	(0.0023)
Oster bounding set $\left[\tilde{\beta },\beta^{*}\right]$	[−0.0486; −0.0442]	[−0.0902; −0.0881]	[−0.3038; 0.2826]	[0.0051; 0.0062]
Bias-adjusted coefficient $\beta^{*}$	−0.0486 **	−0.0881 **	−0.3038 ***	0.0062 **
	[0.0219]	[0.0385]	[0.1042]	[0.0025]
δ statistic	−15.1629	18.9117	−31.8845	−20.2217
**Variables**	**Cardiovascular**	**Heart**	**Cerebrovascular**	**Cancer**
MIDAS coefficient	0.0103	0.0117 *	0.0055	0.0110 *
(clustered S.E.)	(0.0089)	(0.0068)	(0.0073)	(0.0063)
Oster bounding set $\left[\tilde{\beta },\beta^{*}\right]$	[0.0103; 0.0144]	[0.0117; 0.0127]	[0.0055; 0.0073]	[0.0110; 0.0119]
Bias-adjusted coefficient $\beta^{*}$	0.0144 **	0.0127 *	0.0073 **	0.0119 **
	[0.0070]	[0.0074]	[0.0030]	[0.0047]
δ statistic	−11.7525	−22.2240	−3.8717	−15.2021
**Variables**	**Suicide**	**Homicide**	**Accident**	**Pneumonia/Influenza**
MIDAS coefficient	0.0025 ***	−0.0018 **	−0.0087 **	−0.0037 ***
(clustered S.E.)	(0.0007)	(0.0008)	(0.0042)	(0.0013)
Oster bounding set $\left[\tilde{\beta },\beta^{*}\right]$	[0.0025; 0.0032]	[−0.0040; −0.0018]	[−0.0134; −0.0087]	[−0.0037; −0.0032]
Bias-adjusted coefficient $\beta^{*}$	0.0032 ***	−0.0040 ***	−0.0134 ***	−0.0032
	[0.0008]	[0.0014]	[0.0047]	[0.0020]
δ statistic	−4.3146	−2.2227	−6.8234	5.2713

Note: *** *p* < 0.01, ** *p* < 0.05, * *p* < 0.1. The table reports Oster bounds results for the estimated association between EPU and mortality outcomes based on the MIDAS specification (4). The MIDAS coefficient ($\tilde{\beta }$) and its clustered standard error (in parentheses) are shown in the first row of each panel. The Oster bounding set $\left[\tilde{\beta },\beta^{*}\right]$ and the corresponding bias-adjusted estimate $\beta^{*}$, with bootstrapped standard errors (1000 replications) reported in brackets, are presented in the second and third rows of each panel, respectively, under the assumption that δ=1 and $R_{max}=min(1.05\tilde{R},1)$. The bottom row provides the value of δ with β=0 and $R_{max}=min(1.05\tilde{R},1)$. The parameter δ captures the relative importance of unobserved versus observed factors in explaining variation in mortality outcomes, while $R_{max}$ denotes the maximum R-squared value derived from a full regression (5).

**Table 4:** Parameter Estimates of the Determinants of Sex- and Age-specific Mortality Rates.

**Variables**	**Male Mortality**		**Female Mortality**	
	**(1)**	**(2)**	**(1)**	**(2)**
EPU	−0.0626 **	−0.0494 **	−0.0507 **	−0.0416 **
	(0.0261)	(0.0214)	(0.0227)	(0.0210)
Unemployment		−2.3308 **		−1.9635 **
		(1.1471)		(0.8725)
Personal income per capita		−4.4128 ***		−3.0736 ***
		(1.1781)		(0.6399)
$K_{m}$	12	38	24	24
$K_{q}$		15		14
$R^{2}$	0.9739	0.9754	0.9766	0.9782
**Variables**	**15–34-Year-Olds**		**35–54-Year-Olds**	
	**(1)**	**(2)**	**(1)**	**(2)**
EPU	−0.0158 **	−0.0082 **	−0.0510 **	−0.0442 **
	(0.0066)	(0.0038)	(0.0251)	(0.0216)
Unemployment		−1.1916 ***		−2.6414
		(0.4220)		(1.9142)
Personal income per capita		−0.7860 **		−1.6810 *
		(0.3240)		(0.8911)
$K_{m}$	12	14	33	33
$K_{q}$		16		14
$R^{2}$	0.9288	0.9322	0.9709	0.9720
**Variables**	**55–64-Year-Olds**		**≥65-Year-Olds**	
	**(1)**	**(2)**	**(1)**	**(2)**
EPU	−0.1022 *	−0.0902 *	−0.3184 ***	−0.2826 ***
	(0.0611)	(0.0486)	(0.0915)	(0.0875)
Unemployment		3.7420		−10.3572 **
		(2.6734)		(4.7251)
Personal income per capita		−8.4661 ***		−12.9484 ***
		(1.2744)		(4.4452)
$K_{m}$	16	16	24	24
$K_{q}$		8		8
$R^{2}$	0.9776	0.9801	0.9684	0.9695

Note: *** *p* < 0.01, ** *p* < 0.05, * *p* < 0.1. The dependent variables are sex- and age-specific mortality rates. All specifications include state and year fixed effects, demographic controls, and EPU. Column 2 additionally incorporates unemployment and personal income per capita. Standard errors, clustered at the state level, are reported in parentheses.

**Table 5:** Parameter Estimates of the Determinants of Cause-specific Mortality Rates.

**Variables**	**Respiratory**		**Cardiovascular**		**Ischemic Heart**	
	**(1)**	**(2)**	**(1)**	**(2)**	**(1)**	**(2)**
EPU	0.0024	0.0051 **	0.0046	0.0103	0.0101	0.0117 *
	(0.0016)	(0.0023)	(0.0044)	(0.0089)	(0.0067)	(0.0068)
Unemployment		−0.2660 **		−2.0863 ***		−0.4300
		(0.1329)		(0.7393)		(0.3439)
PIPC		−0.6688 ***		−1.9489 ***		−0.7818 ***
		(0.1054)		(0.4680)		(0.2627)
$K_{m}$	24	45	25	37	32	32
$K_{q}$		15		16		16
$R^{2}$	0.9775	0.9794	0.9836	0.9857	0.9568	0.9578
**Variables**	**Cerebrovascular**		**Cancer**		**Suicide**	
	**(1)**	**(2)**	**(1)**	**(2)**	**(1)**	**(2)**
EPU	0.0057 *	0.0055	0.0038 *	0.0110 *	0.0028 ***	0.0025 ***
	(0.0032)	(0.0073)	(0.0021)	(0.0063)	(0.0008)	(0.0007)
Unemployment		−0.6705 **		−0.7782 *		0.0604
		(0.2928)		(0.4101)		(0.0612)
PIPC		−0.3364 *		−0.8848 ***		−0.0396
		(0.1963)		(0.2312)		(0.0348)
$K_{m}$	37	36	12	27	17	15
$K_{q}$		16		16		6
$R^{2}$	0.9207	0.9244	0.9851	0.9862	0.9581	0.9583
**Variables**	**Homicide**		**Accident**		**Pneumonia/Influenza**	
	**(1)**	**(2)**	**(1)**	**(2)**	**(1)**	**(2)**
EPU	−0.0019 **	−0.0018 **	−0.0094 ***	−0.0087 **	−0.0046 **	−0.0037 ***
	(0.0008)	(0.0008)	(0.0036)	(0.0042)	(0.0022)	(0.0013)
Unemployment		−0.1226 ***		−0.9068 **		−0.3328
		(0.0470)		(0.3632)		(0.2071)
PIPC		−0.1323 ***		0.2265 **		−0.0807
		(0.0375)		(0.1143)		(0.0825)
$K_{m}$	14	39	35	48	24	24
$K_{q}$		13		16		13
$R^{2}$	0.9400	0.9417	0.9073	0.9119	0.8633	0.8667

Note: *** *p* < 0.01, ** *p* < 0.05, * *p* < 0.1. The dependent variables are cause-specific mortality rates. All specifications include state and year fixed effects, demographic controls, and EPU. Column 2 additionally incorporates unemployment and personal income per capita (PIPC). Standard errors, clustered at the state level, are reported in parentheses.

**Table 6:** Summary Statistics for Variables Used in the BRFSS Analysis.

Variables	Variable Type	Sample Size	Mean	Std. Dev.
Economic conditions				
EPU	Continuous	5,355,608	188.7	162.3
Unemployment rate (%)	Continuous	5,355,608	6.6	2.7
Risky Health Behaviors				
Current drinker (%)	Dichotomous	5,107,845	53.1	49.9
Binge drinker (%)	Dichotomous	5,057,927	16.4	37.0
Current smoker (%)	Dichotomous	5,190,032	17.0	37.6
Physical inactivity (%)	Dichotomous	5,198,826	24.8	43.2
BMI	Continuous	5,024,720	28.4	8.9
Overweight (%)	Dichotomous	4,984,146	35.5	47.9
Overweight and obese combined (%)	Dichotomous	4,984,146	64.7	47.8
Obese (%)	Dichotomous	4,984,146	29.2	45.5
Self-reported health (%):	Ordinal			
Excellent		5,337,497	19.2	39.3
Very good		5,337,497	32.2	46.7
Good		5,337,497	31.2	46.3
Fair		5,337,497	12.8	33.4
Poor		5,337,497	4.6	21.0
Physical distress (%)	Dichotomous	5,236,785	11.8	32.3
Mental distress (%)	Dichotomous	5,260,921	12.0	32.5
Activity limitation (%)	Dichotomous	2,729,362	15.4	36.1
Individual Characteristics				
Household Income Level (%):	Ordinal			
1 (less than $15,000)		4,495,121	11.2	31.5
2 ($15,000–$25,000)		4,495,121	16.8	37.4
3 ($25,000–$50,000)		4,495,121	24.1	42.8
4 (more than $50,000)		4,495,121	47.9	50.0
Age	Continuous	5,355,608	47.1	17.9
College graduate or higher (%)	Dichotomous	5,332,376	28.2	45.0
Hispanic origin (%)	Dichotomous	5,312,589	15.4	36.1
Female (%)	Dichotomous	5,354,146	51.3	50.0
Married (%)	Dichotomous	5,318,969	52.4	49.9
American Indian or Alaska Native (%)	Dichotomous	5,235,638	2.6	16.0
Black or African American (%)	Dichotomous	5,235,638	12.8	33.4

Note: Data on EPU are collected from www.policyuncertainty.com. Data on unemployment rates are collected from the Federal Reserve Economic Data. Data on risky health behaviors and individual-level characteristics are collected from the Behavioral Risk Factor Surveillance System (BRFSS). The sample covers the period from 2009 to 2020 for the 50 US states and the District of Columbia. BRFSS Sample weights are applied when calculating means and standard deviations.

**Table 7:** Summary Statistics for Variables Used in the ATUS Analysis.

Variables	Variable Type	Sample Size	Mean	Std. Dev.
Economic conditions				
EPU	Continuous	133,723	176.7	132.0
Unemployment rate (%)	Continuous	133,723	6.5	2.5
Time Use Category				
Traveling [18]	Dichotomous	133,723	70.0	76.6
Socializing [12-01, 12-02, 12-03-07, 12-05-01, 12-05-02, 15, 16]	Dichotomous	133,723	69.7	116.8
Sports [13-01, 13-03-01]	Dichotomous	133,723	18.4	54.7
Own Medical [01-03, 08-04]	Dichotomous	133,723	7.7	50.8
Individual Characteristics				
Household Income Level (%):	Ordinal			
1 (less than $15,000)		128,348	10.3	30.4
2 ($15,000–$25,000)		128,348	9.1	28.8
3 ($25,000–$50,000)		128,348	24.4	42.9
4 (more than $50,000)		128,348	56.1	49.6
Age	Continuous	133,723	45.4	18.7
College graduate or higher (%)	Dichotomous	133,723	30.8	46.2
Hispanic origin (%)	Dichotomous	133,723	15.7	36.4
Female (%)	Dichotomous	133,723	51.6	50.0
Married (%)	Dichotomous	133,723	51.9	50.0
American Indian or Alaska Native (%)	Dichotomous	133,723	0.8	8.7
Black or African American (%)	Dichotomous	133,723	12.3	32.8

Note: Data on EPU are collected from www.policyuncertainty.com. Data on unemployment rates are collected from the Federal Reserve Economic Data. Data on time-use categories and individual-level characteristics are collected from the American Time Use Survey (ATUS). Codes in the ATUS lexicon for each activity are shown in square brackets. The sample covers the period from 2009 to 2020 for the 50 US states and the District of Columbia. ATUS Sample weights are applied when calculating means and standard deviations.

**Table 8:** Parameter Estimates of the Determinants of Lifestyle Behaviors.

**Health Behaviors**	**Current Drinker (Probit)**		**Binge Drinker (Probit)**		**Current Smoker (Probit)**		**Physical Inactivity (Probit)**	
	**(1)**	**(2)**	**(1)**	**(2)**	**(1)**	**(2)**	**(1)**	**(2)**
EPU	−0.000047 ***	−0.000035 **	−0.000027 **	−0.000016	−0.000044 ***	−0.000038 ***	−0.000018	−0.000018
	(0.000016)	(0.000014)	(0.000013)	(0.000012)	(0.000012)	(0.000011)	(0.000015)	(0.000014)
Unemployment		−0.0032		−0.0043 **		−0.0039 **		−0.0031
		(0.0034)		(0.0021)		(0.0017)		(0.0024)
Household income		0.2687 ***		0.1149 ***		−0.1784 ***		−0.1866 ***
		(0.0032)		(0.0030)		(0.0041)		(0.0030)
**Health Behaviors**	**BMI (OLS)**		**Overweight (Probit)**		**Overweight/Obese (Probit)**		**Obese (Probit)**	
	**(1)**	**(2)**	**(1)**	**(2)**	**(1)**	**(2)**	**(1)**	**(2)**
EPU	−0.0142 *	−0.0222 ***	−4.53 × 10^−6^	2.78 × 10^−6^	−0.000035 ***	−0.000037 ***	−0.000027 **	−0.000035 ***
	(0.0074)	(0.0077)	(9.19 × 10^−6^)	(9.27 × 10^−6^)	(9.52 × 10^−6^)	(9.74 × 10^−6^)	(0.000011)	(0.000010)
Unemployment		2.9852 *		−0.0012		0.0050 ***		0.0061 ***
		(1.6858)		(0.0010)		(0.0017)		(0.0020)
Household income		−38.2489 ***		0.0537 ***		−0.0121 ***		−0.0691 ***
		(1.3783)		(0.0017)		(0.0022)		(0.0024)
**Health Behaviors**	**Self-assessed health (OProbit)**		**Physical distress (Probit)**		**Mental distress (Probit)**		**Activity limitation (Probit)**	
	**(1)**	**(2)**	**(1)**	**(2)**	**(1)**	**(2)**	**(1)**	**(2)**
EPU	−0.000019 *	−0.000017 **	−0.000012	−6.93 × 10^−6^	7.87 × 10^−6^	4.13 × 10^−6^	−3.74 × 10^−6^	−0.000010
	(9.74 × 10^−6^)	(8.26 × 10^−6^)	(0.000012)	(0.000012)	(0.000016)	(0.000016)	(0.000015)	(0.000014)
Unemployment		−0.0014		−0.0010		0.0043 ***		0.0020
		(0.0017)		(0.0014)		(0.0017)		(0.0021)
Household income		−0.3061 ***		−0.3098 ***		−0.2509 ***		−0.3083 ***
		(0.0044)		(0.0054)		(0.0053)		(0.0044)
**Time Use Category**	**Traveling (OLS)**		**Socializing (OLS)**		**Sports (OLS)**		**Own medical care (OLS)**	
	**(1)**	**(2)**	**(1)**	**(2)**	**(1)**	**(2)**	**(1)**	**(2)**
EPU	−0.0153 ***	−0.0138 ***	0.0079 **	0.0076 *	−0.0012	−0.0014	0.0018	0.0023
	(0.0033)	(0.0032)	(0.0039)	(0.0040)	(0.0025)	(0.0025)	(0.0025)	(0.0025)
Unemployment		−0.7068 **		−0.0337		0.0157		−0.0607
		(0.2801)		(0.4293)		(0.1938)		(0.1897)
Household income		6.3715 ***		−1.2400 ***		2.0497 ***		−2.0318 ***
		(0.2496)		(0.3712)		(0.1619)		(0.1771)

Abbreviations: OProbit, Ordered probit model; Probit, Probit model; OLS, Ordinary least squares. Note: *** *p* < 0.01, ** *p* < 0.05, * *p* < 0.1. The dependent variables are indicators of risky health behaviors. All specifications control for state and year fixed effects, EPU, and individual-level demographic variables, including age, gender, race (American Indian or Alaska Native, Black or African American), education (college graduate), marital status, and ethnicity (Hispanic origin). Column 2 additionally includes unemployment rates and household income percentiles. Ordered probit models are used for ordinal outcomes, probit models for binary outcomes, and OLS models for continuous outcomes. Standard errors, clustered at the state level, are reported in parentheses.

**Table 9:** Long-term effects of Economic Variables on Lifestyle Behaviors.

**Health Behaviors**	**Current Drinker (Probit)**	**Binge Drinker (Probit)**	**Current Smoker (Probit)**	**Physical Inactivity (Probit)**
EPU	−0.000082 **	−0.000037 **	−0.000077 ***	−0.000049
	(0.000038)	(0.000017)	(0.000028)	(0.000032)
Unemployment	0.0030	0.00027	−0.0048 **	−0.0029
	(0.0025)	(0.0020)	(0.0021)	(0.0024)
Household income	0.2689 ***	0.1150 ***	−0.1783 ***	−0.1867 ***
	(0.0032)	(0.0031)	(0.0040)	(0.0030)
**Health Behaviors**	**BMI (OLS)**	**Overweight (Probit)**	**Overweight/Obese (Probit)**	**Obese (Probit)**
EPU	−0.0362 **	−3.30 × 10^−6^	−0.000057 ***	−0.000045 **
	(0.0173)	(0.000017)	(0.000021)	(0.000021)
Unemployment	−0.5779	0.0004	−0.0018	−0.0022
	(1.4509)	(0.0010)	(0.0015)	(0.0014)
Household income	−38.3312 ***	0.0543 ***	−0.0019	−0.0598 ***
	(1.3795)	(0.0016)	(0.0018)	(0.0018)
**Health Behaviors**	**Self-assessed health (OProbit)**	**Physical distress (Probit)**	**Mental distress (Probit)**	**Activity limitation (Probit)**
EPU	−0.000040 **	−0.000041 **	0.000018	−5.04 × 10^−6^
	(0.000020)	(0.000020)	(0.000031)	(0.000027)
Unemployment	−0.0029 **	−0.0033 **	−0.0007	−0.0038 *
	(0.0014)	(0.0013)	(0.0019)	(0.0022)
Household income	−0.3121 ***	−0.3100 ***	−0.2513 ***	−0.3088 ***
	(0.0045)	(0.0055)	(0.0053)	(0.0044)
**Time Use Category**	**Traveling (OLS)**	**Socializing (OLS)**	**Sports (OLS)**	**Own Medical (OLS)**
EPU	−0.0211 ***	0.0095	−0.0049	0.0027
	(0.0073)	(0.0072)	(0.0044)	(0.0057)
Unemployment	0.0405	−0.3548	−0.0817	0.3066
	(0.2651)	(0.4099)	(0.1747)	(0.2143)
Household income	6.3897 ***	−1.2536 ***	2.0525 ***	−2.2387 ***
	(0.2528)	(0.3684)	(0.1669)	(0.1839)

Abbreviations: OProbit, Ordered probit model; Probit, Probit model; OLS, Ordinary least squares. Note: *** *p* < 0.01, ** *p* < 0.05, * *p* < 0.1. The dependent variables are indicators of lifestyle behaviors. All specifications control for state and year fixed effects, individual-level demographic variables including household income, age, gender, race (American Indian or Alaska Native, Black or African American), education (college graduate), marital status, and ethnicity (Hispanic origin), unemployment rates, and EPU. Ordered probit models are used for ordinal outcomes, probit models for binary outcomes, and OLS models for continuous outcomes. Standard errors, clustered at the state level, are reported in parentheses.

**Table 10:** Robustness to Model Specification.

**Variables**	**With Time Trends (1)**	**Pre-2019 Sample (2)**	**Lagged EPU (3)**
EPU	−0.0231 **	−0.0407 **	−0.0515 **
	(0.0105)	(0.0204)	(0.0250)
Unemployment	−6.0835 **	−2.8293	−2.1301 *
	(2.6075)	(2.7023)	(1.2730)
Personal income per capita	−3.1548 ***	−3.7894 ***	−3.5976 ***
	(0.8976)	(1.1116)	(1.0075)
$K_{m}$	39	47	24
$K_{q}$	12	15	12
**Variables**	**Lead EPU (4)**	**Dynamic GMM (5)**	
EPU	−0.0281	−0.0442 **	
	(0.0189)	(0.0209)	
Unemployment	−2.0247 ***	−0.8460	
	(0.6502)	(0.8562)	
Personal income per capita	−3.1326 **	−3.0720 ***	
	(1.5237)	(0.7495)	
Lagged mortality		0.2140 ***	
		(0.0796)	
$K_{m}$	36	24	
$K_{q}$	15	12	

Note: *** *p* < 0.01, ** *p* < 0.05, * *p* < 0.1. The dependent variables are age-adjusted total mortality rates. All specifications include control for state and year fixed effects, demographic variables, unemployment, personal income per capita, and EPU. Column (1) includes state-specific time trends. Column (2) restricts the sample to the pre-2019 period. Column (3) replaces EPU with its one-year lag. Column (4) reports a placebo test using the one-year lead of EPU. Column (5) includes a lagged dependent variable to account for potential persistence in mortality and is estimated using a dynamic panel GMM approach. All specifications include state and year fixed effects. Standard errors, clustered at the state level, are reported in parentheses.

**Table 11:** Robustness to Alternative Lag Polynomials and EPU Indices.

Variables	Exponential Polynomials (1)	EPU_National (2)	EPU_State (3)
EPU	−0.0335 **	−0.0370 ***	−0.0605 **
	(0.0162)	(0.0101)	(0.0262)
Unemployment	−2.2086 **	−2.3003 **	−2.3769 **
	(0.9190)	(0.9105)	(1.0470)
Personal income per capita	−3.7565 ***	−3.7872 ***	−3.6421 ***
	(1.0361)	(0.9605)	(1.0542)
$K_{m}$	37	37	38
$K_{q}$	14	14	14

Note: *** *p* < 0.01, ** *p* < 0.05, * *p* < 0.1. The dependent variables are age-adjusted total mortality rates. All specifications include control for state and year fixed effects, demographic variables, unemployment, personal income per capita, and EPU. Column (1) uses Exponential Almon lag polynomials. Columns (2) and (3) use two alternative EPU indices. Standard errors, clustered at the state level, are reported in parentheses.

**Table 12:** Robustness to Alternative Maximum Lag Horizons.

Variables	Max Lag = 2 Years (1)	Max Lag = 6 Years (2)
EPU	−0.0404 **	−0.0454 **
	(0.0204)	(0.0185)
Unemployment	−2.1140 **	−1.2218
	(0.8296)	(0.9566)
Personal income per capita	−1.3451 **	−3.7720 ***
	(0.5387)	(0.8467)
$K_{m}$	24	50
$K_{q}$	4	17

Notes: *** *p* < 0.01, ** *p* < 0.05, * *p* < 0.1. The dependent variable is the age-adjusted total mortality rate. All specifications include state and year fixed effects, demographic controls, unemployment rates, personal income per capita, and EPU. Column (1) imposes a 2-year maximum lag window, and Column (2) imposes a 6-year maximum lag window. Within each specification, the optimal lag length is selected using the Akaike Information Criterion (AIC). Standard errors, clustered at the state level, are reported in parentheses.

## Data Availability

The data presented in this study were derived from the following resources, available in the public domain: the CDC WONDER database (https://wonder.cdc.gov/, accessed on 10 March 2026), the Economic Policy Uncertainty Index (https://www.policyuncertainty.com/, accessed on 10 March 2026), the Federal Reserve Economic Data (FRED) (https://fred.stlouisfed.org/, accessed on 10 March 2026), the American Community Survey (ACS) (https://www.census.gov/programs-surveys/acs/, accessed on 10 March 2026), the American Time Use Survey (ATUS) (https://www.bls.gov/tus/, accessed on 10 March 2026), and the Behavioral Risk Factor Surveillance System (BRFSS) (https://www.cdc.gov/brfss/, accessed on 10 March 2026).

## Appendix A

**Table A1:** Diagnostic Test Results for Heteroskedasticity, Serial Correlation, and Multicollinearity in the MIDAS Model of Total Mortality Rates.

Test Category	Test	Null Hypothesis	Test Statistic	Result
Heteroskedasticity	Modified Wald test	Homoskedasticity	1942.81 ***	Reject $H_{0}$
Serial Correlation	Wooldridge test	No first-order autocorrelation	17.886 ***	Reject $H_{0}$
Multicollinearity	VIF (EPU)		1.12	
	VIF (Unemployment)		1.47	
	VIF (Personal income per capita)		4.88	
	Mean VIF		3.06	No concern

Note: *** *p* < 0.01, ** *p* < 0.05, * *p* < 0.1. The Modified Wald test examines groupwise heteroskedasticity in the fixed-effects model, with the null hypothesis of homoskedasticity. The Wooldridge test assesses first-order autocorrelation in panel data, with the null hypothesis of no serial correlation. Variance inflation factors (VIFs) are reported to evaluate multicollinearity. A mean VIF below 10 suggests that multicollinearity is not a concern.

**Table A2:** Distributed Lag Model Estimates under Alternative Lag Specifications for Total Mortality Rates.

Variables	(1)	(2)	(3)
EPU	−0.0354 *	−0.0350 *	−0.0342 *
	(0.0208)	(0.0201)	(0.0186)
Un	−0.2944	0.0273	0.4836
	(1.8794)	(2.1938)	(2.2549)
PIPC	0.9527	0.6509	0.6859
	(0.9481)	(1.0058)	(1.0744)
L1.EPU	−0.0031	−0.0114	−0.0138
	(0.0264)	(0.0251)	(0.0259)
L1.Un	−1.4922	−0.0083	0.0855
	(1.6338)	(2.7137)	(2.6047)
L1.PIPC	−3.1534 ***	−0.9784	−1.3970
	(0.9916)	(1.1630)	(1.2781)
L2.EPU		0.0209	0.0201
		(0.0173)	(0.0176)
L2.Un		−2.2003	−1.5618
		(1.4945)	(1.2666)
L2.PIPC		−2.7758 ***	−0.9506
		(0.8728)	(0.8088)
L3.EPU			0.0059
			(0.0232)
L3.Un			−1.1151
			(1.1152)
L3.PIPC			−2.1952 ***
			(0.7270)
$R^{2}$	0.8305	0.8346	0.8368

Note: *** *p* < 0.01, ** *p* < 0.05, * *p* < 0.1. Un denotes the unemployment rate, and PIPC denotes personal income per capita. The dependent variable is the age-adjusted total mortality rate. All specifications include state and year fixed effects and demographic controls. Annual counterparts of EPU, unemployment, and personal income per capita are constructed by averaging their monthly or quarterly values within each year. Standard errors clustered at the state level are reported in parentheses.

**Table A3:** Sensitivity Analysis of Oster Bounds to Alternative Values of δ and *R_max_*.

δ=1	$R_{max}=0.985$	$R_{max}=0.99$	$R_{max}=0.995$	$R_{max}=1$
MIDAS coefficient	−0.0372 **	−0.0372 **	−0.0372 **	−0.0372 **
(clustered S.E.)	(0.0178)	(0.0178)	(0.0178)	(0.0178)
$\mathrm{Oster}\;\mathrm{bounding}\;\mathrm{set}\;[\tilde{\beta },\beta^{*}]$	[−0.0403; −0.0372]	[−0.0435; −0.0372]	[−0.0468; −0.0372]	[−0.0502; −0.0372]
$\mathrm{Bias}-\mathrm{adjusted}\;\mathrm{coefficient}\;\beta^{*}$	−0.0403 **	−0.0435 **	−0.0468 **	−0.0502 **
	[0.0203]	[0.0210]	[0.0229]	[0.0245]
δ statistic	−13.3462	−6.9757	−4.7218	−3.5688
δ=0.5	$R_{max}=0.985$	$R_{max}=0.99$	$R_{max}=0.995$	$R_{max}=1$
MIDAS coefficient	−0.0372 **	−0.0372 **	−0.0372 **	−0.0372 **
(clustered S.E.)	(0.0178)	(0.0178)	(0.0178)	(0.0178)
$\mathrm{Oster}\;\mathrm{bounding}\;\mathrm{set}\;[\tilde{\beta },\beta^{*}]$	[−0.0388; −0.0372]	[−0.0403; −0.0372]	[−0.0419; −0.0372]	[−0.0435; −0.0372]
$\mathrm{Bias}-\mathrm{adjusted}\;\mathrm{coefficient}\;\beta^{*}$	−0.0388 **	−0.0403 **	−0.0419 **	−0.0435 **
	[0.0184]	[0.0198]	[0.0212]	[0.0218]
δ statistic	−13.3462	−6.9757	−4.7218	−3.5688

Note: *** *p* < 0.01, ** *p* < 0.05, * *p* < 0.1. The table reports Oster bounds results for the estimated association between EPU and total mortality based on the MIDAS specification (4). The MIDAS coefficient ($\tilde{\beta }$) and its clustered standard error (in parentheses) are shown in the first row of each panel. The Oster bounding set $\left[\tilde{\beta },\beta^{*}\right]$ and the corresponding bias-adjusted estimate $\beta^{*}$, with bootstrapped standard errors (1000 replications) reported in brackets, are presented in the second and third rows of each panel, respectively, under alternative assumptions on δ and $R_{max}$. The bottom row provides the value of δ, with β=0 and alternative values of $R_{max}$. The parameter δ captures the relative importance of unobserved versus observed factors in explaining variation in the mortality outcome, while $R_{max}$ denotes the maximum R-squared value derived from a full regression (5).

## References

[1] Selmi R., Bouoiyour J. (2019). The financial costs of political uncertainty: Evidence from the 2016 US presidential elections. Scott. J. Political Econ..

[2] Antonakakis N., Gupta R. (2017). Is economic policy uncertainty related to suicide rates? Evidence from the United States. Soc. Indic. Res..

[3] Vandoros S., Avendano M., Kawachi I. (2019). The association between economic uncertainty and suicide in the short-run. Soc. Sci. Med..

[4] Vandoros S., Kawachi I. (2021). Economic uncertainty and suicide in the United States. Eur. J. Epidemiol..

[5] Abdou R., Cassells D., Berrill J., Hanly J. (2022). Revisiting the relationship between economic uncertainty and suicide: An alternative approach. Soc. Sci. Med..

[6] Vandoros S., Avendano M., Kawachi I. (2018). The short-term impact of economic uncertainty on motor vehicle collisions. Prev. Med..

[7] Kanavos P., Vandoros S. (2023). Road traffic mortality and economic uncertainty: Evidence from the United States. Soc. Sci. Med..

[8] Kyriopoulos I., Nikoloski Z., Mossialos E. (2019). Does economic recession impact newborn health? Evidence from Greece. Soc. Sci. Med..

[9] Kawachi I., Kyriopoulos I., Vandoros S. (2023). Economic uncertainty and cardiovascular disease mortality. Health Econ..

[10] Kyriopoulos I., Vandoros S., Kawachi I. (2023). State-level economic uncertainty and cardiovascular disease deaths: Evidence from the United States. Eur. J. Epidemiol..

[11] Huang W., Lei X., Yu M. (2024). Economic policy uncertainty, health status, and mortality. Soc. Sci. Med..

[12] Baker S.R., Bloom N., Davis S.J. (2016). Measuring economic policy uncertainty. Q. J. Econ..

[13] Ruhm C.J. (2000). Are recessions good for your health?. Q. J. Econ..

[14] Ruhm C.J. (1995). Economic conditions and alcohol problems. J. Health Econ..

[15] Miller D.L., Page M.E., Stevens A.H., Filipski M. (2009). Why are recessions good for your health?. Am. Econ. Rev..

[16] Laporte A. (2004). Do economic cycles have a permanent effect on population health? Revisiting the Brenner hypothesis. Health Econ..

[17] Brenner M.H. (2005). Commentary: Economic growth is the basis of mortality rate decline in the 20th century–experience of the United States 1901–2000. Int. J. Epidemiol..

[18] Bhalotra S. (2010). Fatal fluctuations? Cyclicality in infant mortality in India. J. Dev. Econ..

[19] Botha F., Nguyen V.H. (2022). Opposite nonlinear effects of unemployment and sentiment on male and female suicide rates: Evidence from Australia. Soc. Sci. Med..

[20] Nicolini E.A. (2007). Was Malthus right? A VAR analysis of economic and demographic interactions in pre-industrial England. Eur. Rev. Econ. Hist..

[21] Milani F. (2021). COVID-19 outbreak, social response, and early economic effects: A global VAR analysis of cross-country interdependencies. J. Popul. Econ..

[22] Bianchi F., Bianchi G., Song D. (2023). The long-term impact of the COVID-19 unemployment shock on life expectancy and mortality rates. J. Econ. Dyn. Control.

[23] Ghysels E., Sinko A., Valkanov R. (2007). MIDAS regressions: Further results and new directions. Econom. Rev..

[24] Brenner M.H. (1973). Mental Illness and the Economy.

[25] Brenner M.H. (1987). Economic change, alcohol consumption and heart disease mortality in nine industrialized countries. Soc. Sci. Med..

[26] Brenner M.H. (1975). Trends in alcohol consumption and associated illnesses. Some effects of economic changes. Am. J. Public Health.

[27] Brenner M.H. (1979). Mortality and the national economy: A review, and the experience of England and Wales, 1936–1976. Lancet.

[28] Gerdtham U.-G., Johannesson M. (2005). Business cycles and mortality: Results from Swedish microdata. Soc. Sci. Med..

[29] Browning M., Heinesen E. (2012). Effect of job loss due to plant closure on mortality and hospitalization. J. Health Econ..

[30] McInerney M., Mellor J.M. (2012). Recessions and seniors’ health, health behaviors, and healthcare use: Analysis of the Medicare Current Beneficiary Survey. J. Health Econ..

[31] Laliotis I., Stavropoulou C. (2018). Crises and mortality: Does the level of unemployment matter?. Soc. Sci. Med..

[32] Marshall J.R., Funch D.P. (1979). Mental illness and the economy: A critique and partial replication. J. Health Soc. Behav..

[33] Forbes J.F., McGregor A. (1984). Unemployment and mortality in post-war Scotland. J. Health Econ..

[34] Wagstaff A. (1985). Time series analysis of the relationship between unemployment and mortality: A survey of econometric critiques and replications of Brenner’s studies. Soc. Sci. Med..

[35] Lee C., Kim K. (2017). Changing relationship between unemployment and mortality in South Korea. Health Econ..

[36] Ruhm C.J. (2015). Recessions, healthy no more?. J. Health Econ..

[37] Ruhm C.J. (2016). Health effects of economic crises. Health Econ..

[38] Heutel G., Ruhm C.J. (2016). Air pollution and procyclical mortality. J. Assoc. Environ. Resour. Econ..

[39] Neumayer E. (2004). Recessions lower (some) mortality rates: Evidence from Germany. Soc. Sci. Med..

[40] Haaland V.F., Telle K. (2015). Pro-cyclical mortality across socioeconomic groups and health status. J. Health Econ..

[41] Granados J.A.T. (2005). Recessions and mortality in Spain, 1980–1997. Eur. J. Popul. /Rev. Eur. Démogr..

[42] Cervini-Plá M., Vall-Castelló J. (2021). Business cycle and mortality in Spain. Eur. J. Health Econ..

[43] Van den Berg G.J., Gerdtham U.-G., von Hinke S., Lindeboom M., Lissdaniels J., Sundquist J., Sundquist K. (2017). Mortality and the business cycle: Evidence from individual and aggregated data. J. Health Econ..

[44] Gerdtham U.-G., Ruhm C.J. (2006). Deaths rise in good economic times: Evidence from the OECD. Econ. Hum. Biol..

[45] Granados J.A.T., Ionides E.L. (2017). Population health and the economy: Mortality and the great recession in Europe. Health Econ..

[46] Ariizumi H., Schirle T. (2012). Are recessions really good for your health? evidence from Canada. Soc. Sci. Med..

[47] Ferrie J.E., Shipley M.J., Newman K., Stansfeld S.A., Marmot M. (2005). Self-reported job insecurity and health in the Whitehall II study: Potential explanations of the relationship. Soc. Sci. Med..

[48] Burgard S.A., Brand J.E., House J.S. (2009). Perceived job insecurity and worker health in the United States. Soc. Sci. Med..

[49] László K.D., Pikhart H., Kopp M.S., Bobak M., Pajak A., Malyutina S., Salavecz G., Marmot M. (2010). Job insecurity and health: A study of 16 European countries. Soc. Sci. Med..

[50] Caroli E., Godard M. (2016). Does job insecurity deteriorate health?. Health Econ..

[51] Kalleberg A.L. (2018). Precarious Lives: Job Insecurity and Well-Being in Rich Democracies.

[52] Bridges S., Disney R. (2010). Debt and depression. J. Health Econ..

[53] Rohde N., Tang K.K., Osberg L., Rao D.S.P. (2017). Is it vulnerability or economic insecurity that matters for health?. J. Econ. Behav. Organ..

[54] Odle-Dusseau H.N., Matthews R.A., Wayne J.H. (2018). Employees’ financial insecurity and health: The underlying role of stress and work–family conflict appraisals. J. Occup. Organ. Psychol..

[55] Abdou R., Cassells D., Berrill J., Hanly J. (2020). An empirical investigation of the relationship between business performance and suicide in the US. Soc. Sci. Med..

[56] Liu L. (2023). Economic uncertainty and population health: Insights from emerging markets and developing countries. Front. Public Health.

[57] Powdthavee N., Plagnol A.C., Frijters P., Clark A.E. (2019). Who got the Brexit blues? The effect of Brexit on subjective wellbeing in the UK. Economica.

[58] Trougakos J.P., Chawla N., McCarthy J.M. (2020). Working in a pandemic: Exploring the impact of COVID-19 health anxiety on work, family, and health outcomes. J. Appl. Psychol..

[59] Kavetsos G., Kawachi I., Kyriopoulos I., Vandoros S. (2021). The effect of the Brexit referendum result on subjective well-being. J. R. Stat. Soc. Ser. A Stat. Soc..

[60] Rettie H., Daniels J. (2021). Coping and tolerance of uncertainty: Predictors and mediators of mental health during the COVID-19 pandemic. Am. Psychol..

[61] de Bruin A., Agyemang A., Chowdhury M.I.H. (2020). New insights on suicide: Uncertainty and political conditions. Appl. Econ. Lett..

[62] Claveria O. (2022). Global economic uncertainty and suicide: Worldwide evidence. Soc. Sci. Med..

[63] Er S.T., Demir E., Sari E. (2023). Suicide and economic uncertainty: New findings in a global setting. SSM-Popul. Health.

[64] Carroll C.D. (1994). How does future income affect current consumption?. Q. J. Econ..

[65] Carroll C.D. (1997). Buffer-stock saving and the life cycle-permanent income hypothesis. Q. J. Econ..

[66] Coibion O., Georgarakos D., Gorodnichenko Y., Kenny G., Weber M. (2024). The effect of macroeconomic uncertainty on household spending. Am. Econ. Rev..

[67] Dixit A.K. (1994). Investment Under Uncertainty.

[68] Leahy J.V., Whited T.M. (1996). The effect of uncertainty on investment: Some stylized trends. J. Money Credit Bank..

[69] Bloom N., Bond S., Van Reenen J. (2007). Uncertainty and investment dynamics. Rev. Econ. Stud..

[70] Bloom N. (2009). The impact of uncertainty shocks. Econometrica.

[71] Handley K., Limao N. (2015). Trade and investment under policy uncertainty: Theory and firm evidence. Am. Econ. J. Econ. Policy.

[72] Handley K., Limão N. (2017). Policy uncertainty, trade, and welfare: Theory and evidence for China and the United States. Am. Econ. Rev..

[73] Bloom N. (2014). Fluctuations in uncertainty. J. Econ. Perspect..

[74] Kalcheva I., McLemore P., Sias R. (2021). Economic policy uncertainty and self-control: Evidence from unhealthy choices. J. Financ. Quant. Anal..

[75] Dragouni M., Filis G., Gavriilidis K., Santamaria D. (2016). Sentiment, mood and outbound tourism demand. Ann. Tour. Res..

[76] Demir E., Gozgor G. (2018). Does economic policy uncertainty affect tourism?. Ann. Tour. Res..

[77] Abadie A., Athey S., Imbens G.W., Wooldridge J.M. (2023). When should you adjust standard errors for clustering?. Q. J. Econ..

[78] Ruhm C.J. (2019). Drivers of the fatal drug epidemic. J. Health Econ..

[79] Oster E. (2019). Unobservable selection and coefficient stability: Theory and evidence. J. Bus. Econ. Stat..

[80] Altonji J.G., Elder T.E., Taber C.R. (2005). Selection on observed and unobserved variables: Assessing the effectiveness of Catholic schools. J. Political Econ..

[81] Black B., De Carvalho A.G., Khanna V., Kim W., Yurtoglu B. (2014). Methods for multicountry studies of corporate governance: Evidence from the BRIKT countries. J. Econom..

[82] Nunn N., Wantchekon L. (2011). The slave trade and the origins of mistrust in Africa. Am. Econ. Rev..

[83] Baker S.R., Davis S.J., Levy J.A. (2022). State-level economic policy uncertainty. J. Monet. Econ..

[84] Stevens A.H., Miller D.L., Page M.E., Filipski M. (2015). The best of times, the worst of times: Understanding pro-cyclical mortality. Am. Econ. J. Econ. Policy.

[85] Aguiar M., Hurst E., Karabarbounis L. (2013). Time use during the Great Recession. Am. Econ. Rev..

